# Lateral olfactory tract usher substance (LOTUS), an endogenous Nogo receptor antagonist, ameliorates disease progression in amyotrophic lateral sclerosis model mice

**DOI:** 10.1038/s41420-023-01758-7

**Published:** 2023-12-14

**Authors:** Takuya Ikeda, Keita Takahashi, Minatsu Higashi, Hiroyasu Komiya, Tetsuya Asano, Akihiro Ogasawara, Shun Kubota, Shunta Hashiguchi, Misako Kunii, Kenichi Tanaka, Mikiko Tada, Hiroshi Doi, Hideyuki Takeuchi, Kohtaro Takei, Fumiaki Tanaka

**Affiliations:** 1 Department of Neurology and Stroke Medicine, Yokohama City University Graduate School of Medicine, Yokohama, 236-0004 Japan; 2 Molecular Medical Bioscience Laboratory, Yokohama City University Graduate School of Medical Life Science, Yokohama, 236-0004 Japan

**Keywords:** Amyotrophic lateral sclerosis, Cell death in the nervous system

## Abstract

Nogo–Nogo receptor 1 (NgR1) signaling is significantly implicated in neurodegeneration in amyotrophic lateral sclerosis (ALS). We previously showed that lateral olfactory tract usher substance (LOTUS) is an endogenous antagonist of NgR1 that prevents all myelin-associated inhibitors (MAIs), including Nogo, from binding to NgR1. Here we investigated the role of LOTUS in ALS pathogenesis by analyzing G93A-mutated human superoxide dismutase 1 (SOD1) transgenic (Tg) mice, as an ALS model, as well as newly generated LOTUS-overexpressing SOD1 Tg mice. We examined expression profiles of LOTUS and MAIs and compared motor functions and survival periods in these mice. We also investigated motor neuron survival, glial proliferation in the lumbar spinal cord, and neuromuscular junction (NMJ) morphology. We analyzed downstream molecules of NgR1 signaling such as ROCK2, LIMK1, cofilin, and ataxin-2, and also neurotrophins. In addition, we investigated LOTUS protein levels in the ventral horn of ALS patients. We found significantly decreased LOTUS expression in both SOD1 Tg mice and ALS patients. LOTUS overexpression in SOD1 Tg mice increased lifespan and improved motor function, in association with prevention of motor neuron loss, reduced gliosis, increased NMJ innervation, maintenance of cofilin phosphorylation dynamics, decreased levels of ataxin-2, and increased levels of brain-derived neurotrophic factor (BDNF). Reduced LOTUS expression may enhance neurodegeneration in SOD1 Tg mice and ALS patients by activating NgR1 signaling, and in this study LOTUS overexpression significantly ameliorated ALS pathogenesis. LOTUS might serve as a promising therapeutic target for ALS.

## Introduction

Amyotrophic lateral sclerosis (ALS) is an adult-onset neurodegenerative disease characterized by selective degeneration of brain and spinal motor neurons. It leads to progressive muscle weakness, amyotrophy, and eventual death from respiratory paralysis within a few years after onset.

Although the precise mechanisms of ALS pathogenesis have yet to be elucidated, Nogo, a representative myelin-associated inhibitor (MAI), and Nogo receptor-1 (NgR1) have recently attracted attention as molecules potentially implicated in ALS [1]. Other MAIs include myelin-associated glycoprotein (MAG), oligodendrocyte myelin glycoprotein (OMgp), and B-lymphocyte stimulator (BLyS)/B-cell activating factor (BAFF), all of which are mainly expressed by glial cells. MAIs suppress axonal outgrowth and neuronal regeneration in the central nervous system (CNS) by binding to NgR1 on neurons and their axons [2–5]. Recent studies have reported that another NgR1 ligand, chondroitin sulfate proteoglycan (CSPG), is expressed in glial scars, and like MAIs, it inhibits axon growth and promotes neurodegeneration [6, 7]. Thus, blocking the binding of MAIs and CSPG to NgR1 might be neuroprotective, and this therapeutic strategy was found to be effective in various models of acute and chronic CNS disorders [8–10]. In ALS, MAIs, and CSPGs have been considered to induce axonal degeneration through NgR1 signaling pathways [11–14] and may be good therapeutic targets. In fact, previous studies showed that expression of Nogo-A, a Nogo isoform, was enhanced in the muscles of ALS patients [15], and genetic deletion of Nogo-A in ALS model mice harboring a mutant human superoxide dismutase 1 (SOD1) transgene prolonged lifespan, suppressed muscle denervation, increased motor neuronal survival, and reduced the number of ubiquitinated inclusions [12]. Furthermore, treatment with a neutralizing antibody against Nogo-A suppressed disease progression in G93A human SOD1 transgenic (Tg) mice [16]. In spinal cord–injured mice, however, triple-gene deletion of Nogo‐A, MAG, and OMgp was more effective than Nogo-A single-gene deletion in terms of increasing axonal growth and improving locomotion [17]. Therefore, targeting multiple MAIs might be ideal in the development of novel ALS therapeutics.

We previously identified lateral olfactory tract usher substance (LOTUS) as an endogenous NgR1 antagonist that inhibited Nogo–NgR1 binding [18]. We also revealed that LOTUS suppressed not only Nogo–NgR1 binding but also NgR1 binding to all ligands, including MAIs and CSPGs [19], and that it strongly promoted neuronal regeneration and functional recovery in mouse models of spinal cord injury [20–22] and cerebral infarction [23] and in a rat model of unilateral pyramidotomy [24]. In addition, we found that a decreased concentration of LOTUS in human cerebrospinal fluid reflected the severity of neurological disorders associated with axonal degeneration [25, 26]. Thus, we hypothesized that targeting LOTUS might promote axonal regeneration due to its function as a potent NgR1 inhibitor, and that it might therefore suppress ALS progression. Here, we examined the role and therapeutic efficacy of LOTUS in ALS using SOD1 Tg mice.

## Results

### Protein levels of endogenous LOTUS decreased at an earlier stage than those of NgR1 and MAIs in the lumbar spinal cords of SOD1 Tg mice

**Fig. 1:**
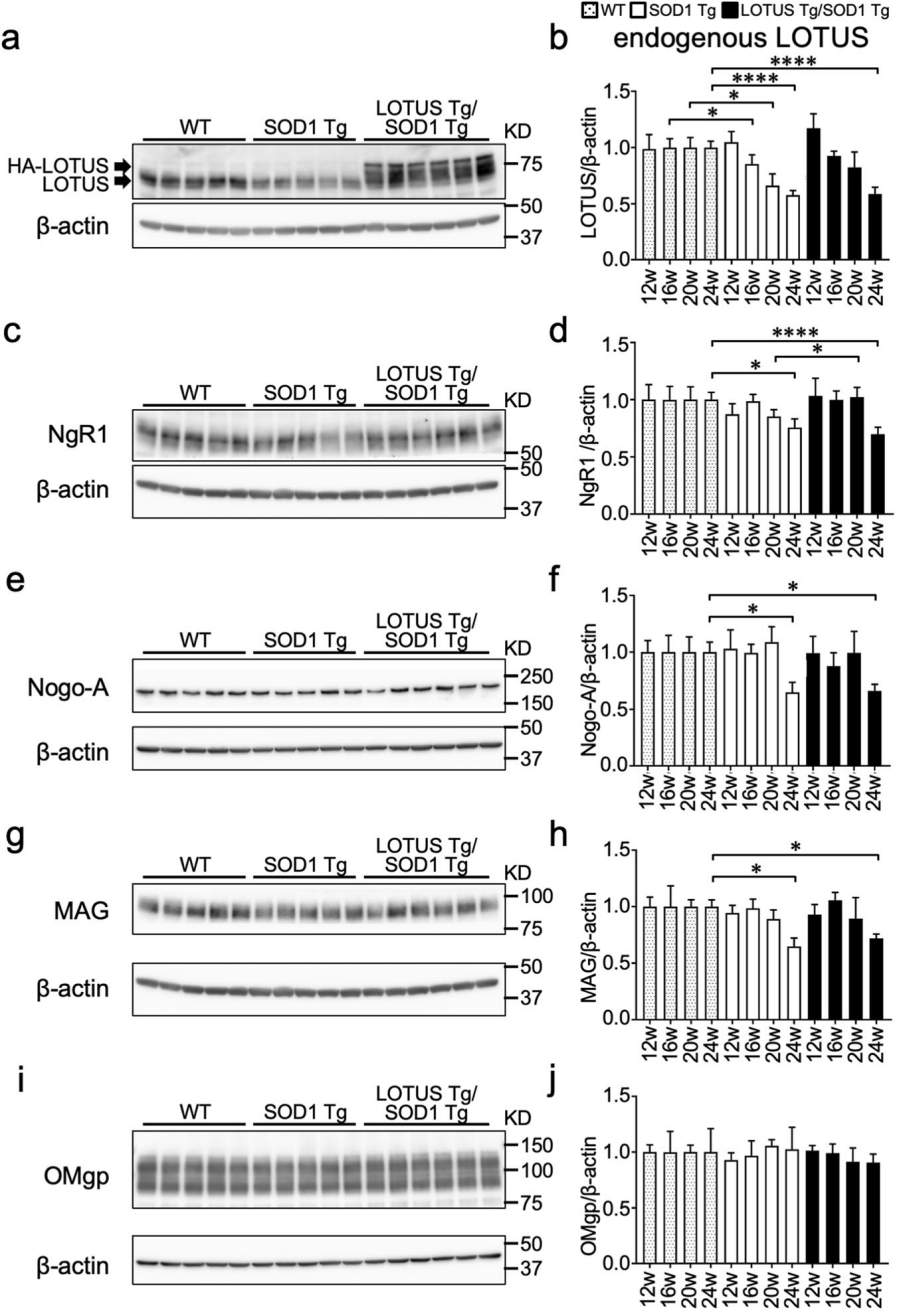
Immunoblotting analyses of LOTUS, NgR1, and MAIs in the lumbar spinal cords of WT, SOD1 Tg, and LOTUS Tg/SOD1 Tg mice. **a** Immunoblots of LOTUS at 20 weeks. HA-LOTUS, overexpressed hemagglutinin-tagged LOTUS; LOTUS, endogenous LOTUS. **b** Quantitative data of (**a**) at 12, 16, 20, and 24 weeks. **c** Immunoblots of NgR1 at 20 weeks. **d** Quantitative data of (**c**) at 12, 16, 20, and 24 weeks. **e** Immunoblots of Nogo-A at 20 weeks. **f** Quantitative data of (**e**) at 12, 16, 20, and 24 weeks. **g** Immunoblots of MAG at 20 weeks. **h** Quantitative data of (**g**) at 12, 16, 20, and 24 weeks. **i** Immunoblots of OMgp at 20 weeks. **j** Quantitative data of (**i**) at 12, 16, 20, and 24 weeks. Values are means ± SD (WT: n = 5; SOD1 Tg: n = 4 (12 weeks) or 5 (16, 20, and 24 weeks); LOTUS Tg/ SOD1 Tg: n = 5 (16 weeks) or 6 (12, 20, and 24 weeks)). *, *p* < 0.05; ****, *p* < 0.0001 (one-way ANOVA followed by post hoc Tukey’s test).

First, we evaluated the protein levels of endogenous LOTUS, NgR1, Nogo-A, MAG, and OMgp in the lumbar spinal cords of wild-type (WT), SOD1 Tg, and LOTUS Tg/SOD1 Tg mice at 12, 16, 20, and 24 weeks. Compared with WT mice, SOD1 Tg mice showed significant and gradual decreases in LOTUS protein levels from 16 weeks to the terminal stage (*p* = 0.021; Fig. 1a, b). LOTUS Tg/SOD1 Tg mice exhibited no declines in LOTUS protein levels at 16 or 20 weeks, but there was a significant decline at 24 weeks (*p* < 0.0001; Fig. 1a, b). At 24 weeks only, the protein levels of NgR1, Nogo-A, and MAG in SOD1 Tg mice and LOTUS Tg/SOD1 Tg mice were significantly lower than those in WT mice (Fig. 1c–h). The protein level of OMgp was not significantly different between WT, SOD1 Tg, and LOTUS Tg/SOD1 Tg mice throughout their lifespan (Fig. 1i, j).

### LOTUS expression was decreased in the lumbar spinal cords of ALS patients

**Fig. 2:**
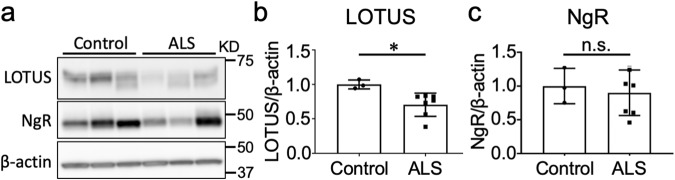
Postmortem LOTUS and NgR expression levels in ALS patients. **a** Representative immunoblots of LOTUS and NgR in the ventral horn of the lumbar spinal cords of postmortem patients with ALS and disease controls. **b** Quantitative data of LOTUS. **c** Quantitative data of NgR. Values are means ± SD (Control: n = 3; ALS: n = 7). *, *p* < 0.05 (Student’s *t*-test).

Next, we investigated whether LOTUS expression in the ventral horn of the lumbar spinal cord was also decreased in ALS patients. Immunoblotting in seven postmortem patients with ALS and three postmortem control patients without spinal involvement (Table S2) revealed that LOTUS expression was significantly lower in ALS patients than in controls (*p* = 0.021; Fig. 2a, b). By contrast, NgR expression was not significantly decreased in ALS patients (*p* = 0.632; Fig. 2a, c), suggesting that reduction of LOTUS expression was not merely a reflection of motor neuronal loss.

### LOTUS improved motor function and survival in SOD1 Tg mice

**Fig. 3:**
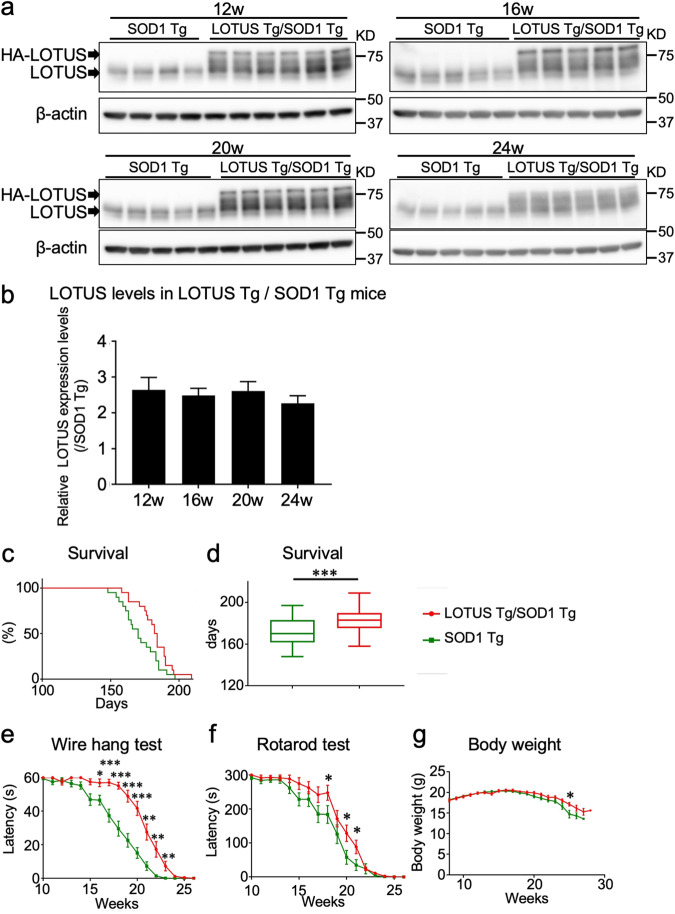
Survival duration and behavioral analyses of SOD1 Tg and LOTUS Tg/SOD1 Tg mice. **a** Immunoblots of LOTUS in the lumbar spinal cords of SOD1 Tg mice and LOTUS Tg/SOD1 Tg mice at 12, 16, 20, and 24 weeks. HA-LOTUS, overexpressed hemagglutinin-tagged LOTUS; LOTUS, endogenous LOTUS. **b** Quantitative data of (**a**). Relative LOTUS overexpression levels: expression ratio of total LOTUS level (endogenous plus transgene) in LOTUS Tg/SOD1 Tg mice to endogenous LOTUS level in SOD1 Tg mice. Comparison of behavioral analyses in SOD1 Tg and LOTUS Tg/SOD1 Tg mice: (**c**) Kaplan–Meier survival curves, (**d**) Survival durations, (**e**) Wire hang test, (**f**) Rotarod test, (**g**) Body weight. Values are means ± SD (Survival: n = 22; Wire hang test, Rotarod test, Body weight: n = 20). *, *p* < 0.05; **, *p* < 0.01; ***, *p* < 0.001; ****, *p* < 0.0001 (Gehan–Breslow–Wilcoxon test and Student’s *t*-test).

Since LOTUS expression in the lumbar ventral horn was decreased not only in SOD1 Tg mice but also in ALS patients, we next attempted to overexpress LOTUS in the SOD1 mice. The total expression level of LOTUS (endogenous plus transgene) in LOTUS Tg/SOD1 Tg mice was approximately 2.5-fold higher than that of endogenous LOTUS in SOD1 Tg mice throughout the lifespan (*p* < 0.0001; Fig. 3a, b). In terms of phenotypic and behavioral differences, LOTUS Tg/SOD1 Tg mice exhibited significantly longer survival than SOD1 Tg mice (183.2 ± 2.7 days *vs*. 171.8 ± 2.7 days, respectively, *p* = 0.0045; Fig. 3c, d), and LOTUS Tg/SOD1 Tg mice had significantly superior performance on the wire hang and Rotarod tests. At 25 weeks only, LOTUS Tg/SOD1 Tg mice had a significantly higher body weight than SOD1 Tg mice. These data suggested the beneficial effects of LOTUS on ALS progression.

### LOTUS overexpression suppressed motor neuronal death, gliosis, and axonal degeneration

**Fig. 4:**
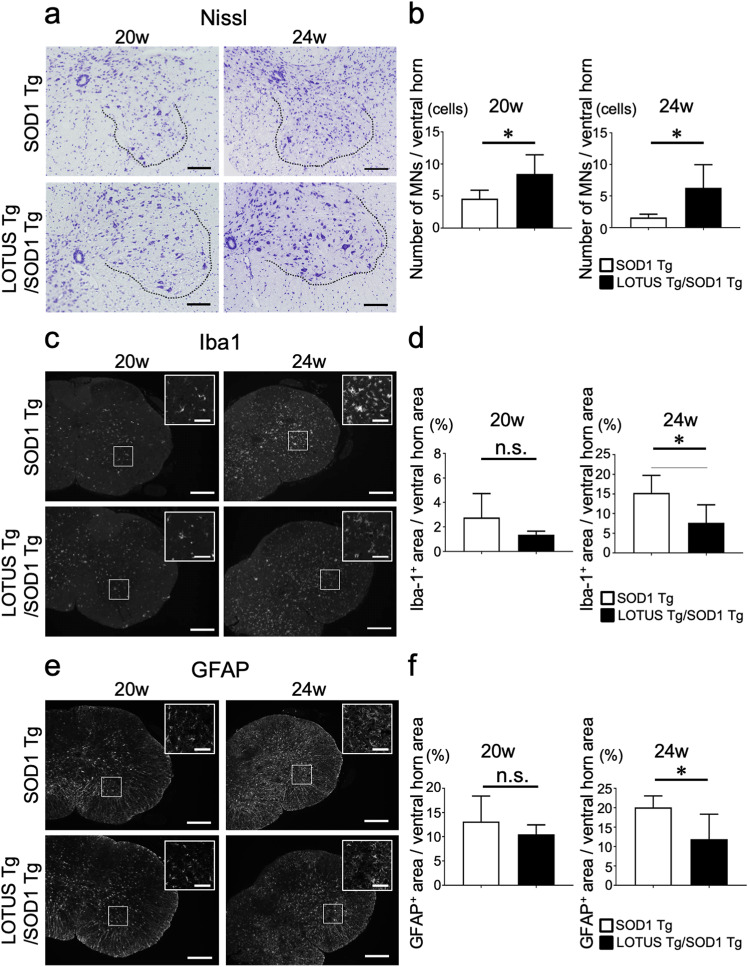
Histological analyses of the lumbar spinal cords of SOD1 Tg and LOTUS Tg/SOD1 Tg mice. **a** Representative micrographs of Nissl staining in the ventral horn of lumbar spinal cords. Scale bars, 100 μm. **b** Quantitative data of surviving motor neurons (MNs). **c** Representative fluorescence micrographs of anti-Iba1 immunostaining in lumbar spinal cords. Scale bars, 200 μm and 50 μm in insets. **d** Quantitative data of Iba1-positive areas. **e** Representative fluorescence micrographs of anti-GFAP immunostaining in lumbar spinal cords. Scale bars, 200 μm and 50 μm in insets. **f** Quantitative data of GFAP-positive areas. Values are means ± SD (n = 5). *, *p* < 0.05 (Student’s *t*-test).

We next used Nissl staining to examine the effects of LOTUS overexpression on motor neurons in the lumbar spinal cord. Nissl staining data revealed that LOTUS Tg/SOD1 Tg mice had a significantly higher density of motor neurons in the ventral horn than SOD1 Tg mice, at both 20 and 24 weeks (20 weeks, *p* = 0.029; 24 weeks, *p* = 0.022; Fig. 4a, b).

Because gliosis is an important pathological feature related to the progression of ALS [27, 28], we next evaluated microgliosis and astrogliosis by anti-Iba1 and anti-GFAP immunostaining, respectively. Our results showed that both microgliosis and astrogliosis were significantly more suppressed in LOTUS Tg/SOD1 Tg mice compared with SOD1 Tg mice at 24 weeks (Iba-1, *p* = 0.028; GFAP, *p* = 0.032), whereas no significant differences were observed at 20 weeks (Iba-1, *p* = 0.15; GFAP, *p* = 0.32) (Fig. 4c–f).

**Fig. 5:**
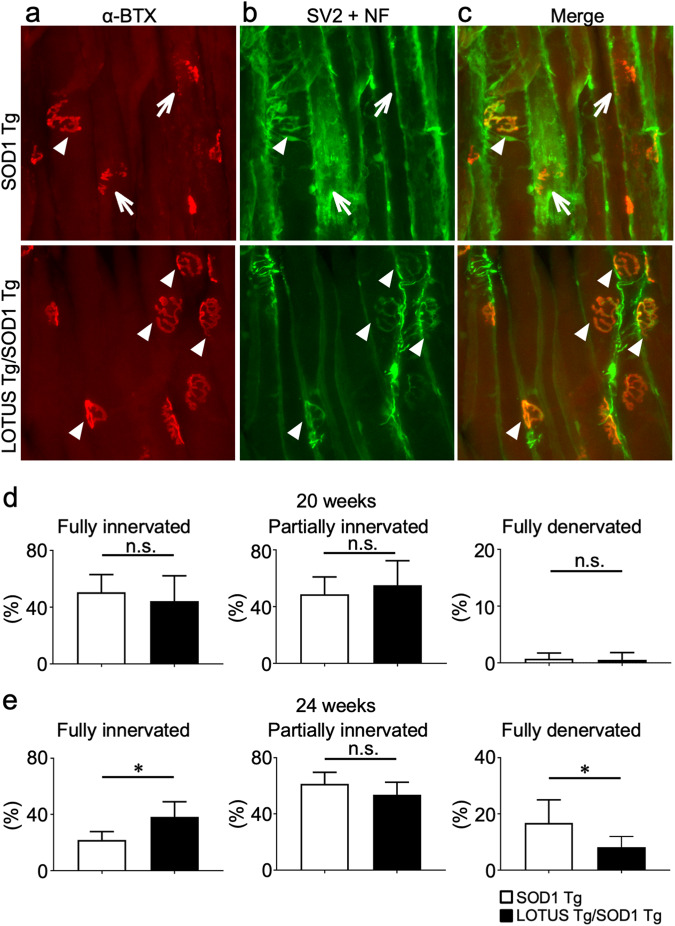
Innervated and denervated NMJs in SOD1 Tg and LOTUS Tg/SOD1 Tg mice. Representative fluorescence micrographs of immunostained NMJs in tibialis anterior muscles at 20 and 24 weeks: (**a**) Postsynaptic acetylcholine receptors (red) stained with α-BTX, (**b**) Nerve terminals (green) stained with neurofilament and synaptic vesicle protein 2 A, (**c**) Merged image of (**a**) and (**b**). Quantitative data of fully innervated, partially innervated, and fully denervated NMJs at 24 weeks (**d**) and at 20 weeks (**e**). Arrowheads show fully innervated NMJs and arrows show fully denervated NMJs. Values are means ± SD (SOD1 Tg: n = 5; LOTUS Tg/SOD1 Tg: n = 6). *, *p* < 0.05 (Student’s *t*-test).

We then assessed axonal degeneration by determining the extent of innervation and denervation in the NMJ. The percentage of fully innervated NMJs was significantly higher in LOTUS Tg/SOD1 Tg mice than in SOD1 Tg mice at 24 weeks (*p* = 0.015; Fig. 5a–c, e). By contrast, the percentage of fully denervated NMJs was significantly lower in LOTUS Tg/SOD1 Tg mice compared with SOD1 Tg mice at the same time point (*p* = 0.045; Fig. 5a–c, e). No significant differences in innervation or denervation were found between the two mouse types at 20 weeks (Fig. 5d).

### LOTUS overexpression partially affected signaling pathways downstream of NgR1

**Fig. 6:**
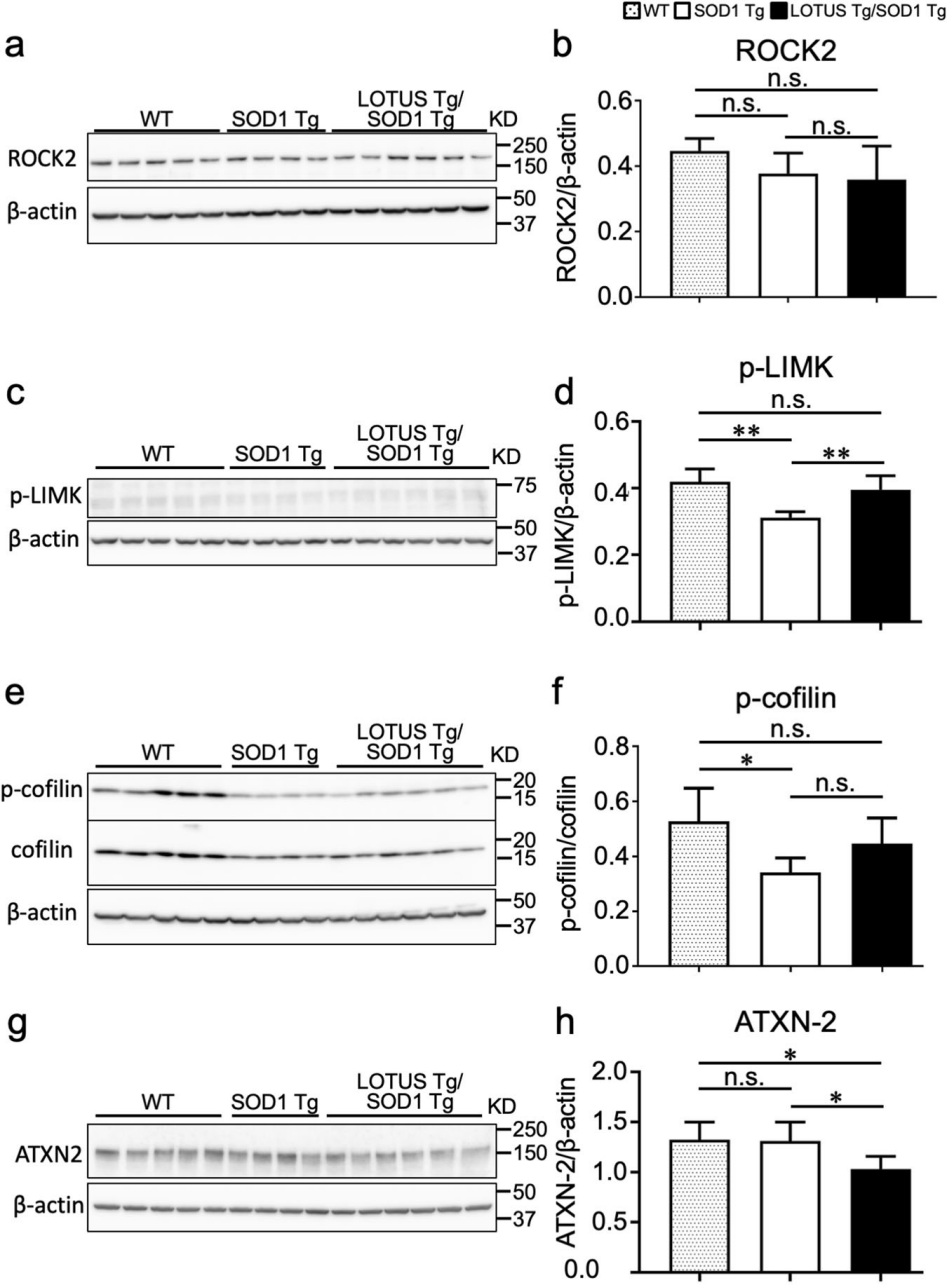
Quantitative analyses of signaling pathways downstream of NgR1 in lumbar spinal cords of WT, SOD1 Tg, and LOTUS Tg/SOD1 Tg mice. **a** Immunoblots of ROCK2 in lumbar spinal cords at 12 weeks. **b** Quantitative data of (**a**). **c** Immunoblots of phosphorylated LIMK1 at 12 weeks. **d** Quantitative data of (**c**). **e** Immunoblots of cofilin and phosphorylated cofilin (p-cofilin) at 12 weeks. **f** Quantitative data of (**e**). **g** Immunoblots of ataxin-2 at 12 weeks. **h** Quantitative data of (**f**). Values are means ± SD (WT: n = 5; SOD1 Tg: n = 4; LOTUS Tg/SOD1 Tg: n = 6). *, *p* < 0.05, **, *p* < 0.01 (one-way ANOVA followed by post hoc Tukey’s test).

We examined the alterations of signaling pathways downstream of NgR1 in the lumbar spinal cords of WT, SOD1 Tg, and LOTUS Tg/SOD1 Tg mice at 12 weeks. Although the protein levels of ROCK2 were similar in all three types of mice (Fig. 6a, b), the phosphorylation levels of LIMK1, a downstream molecule of ROCK2, were significantly decreased in SOD1 Tg mice compared to WT and LOTUS Tg/SOD1 Tg mice (WT *vs*. SOD1 Tg, *p* = 0.0022; SOD1 Tg *vs*. LOTUS Tg/SOD1 Tg, *p* = 0.01; Fig. 6c, d). Furthermore, we investigated the phosphorylation levels of cofilin, which is phosphorylated by LIMK1, and found that they were significantly lower in SOD1 Tg mice than in WT mice (*p* = 0.042; Fig. 6e, f). There was no significant difference between WT and LOTUS Tg/SOD1 Tg mice.

In addition to these conventional molecules, we investigated ataxin-2 protein, a newly identified downstream molecule of NgR1, and found a significant decrease in LOTUS Tg/SOD1 Tg mice compared to WT and SOD1 Tg mice (WT *vs*. LOTUS Tg/SOD1 Tg, *p* = 0.028; SOD1 Tg *vs*. LOTUS Tg/SOD1 Tg, *p* = 0.048; Fig. 6g, h).

We also investigated whether LOTUS overexpression affected the expression levels of neurotrophins in the lumbar spinal cord at 24 weeks. The expression levels of BDNF were significantly higher in LOTUS Tg/SOD1 Tg mice than in WT and SOD1 Tg mice (WT *vs*. LOTUS Tg/SOD1 Tg, *p* = 0.0056; SOD1 Tg *vs*. LOTUS Tg/SOD1 Tg, *p* = 0.0058; Fig. S2). The expression levels of NGF were significantly higher in both SOD1 Tg and LOTUS Tg/SOD1 Tg mice than in WT mice (WT *vs*. SOD1 Tg, *p* = 0.019; WT *vs*. LOTUS Tg/SOD1 Tg, *p* = 0.0092; Fig. S2), whereas there was no significant difference between SOD1 Tg and LOTUS Tg/SOD1 Tg mice (*p* = 0.89). NT-3 expression levels did not differ significantly between WT, SOD1 Tg, and LOTUS Tg/SOD1 Tg mice (Fig. S2).

## Discussion

Signaling pathways downstream of NgR1 that are activated by CSPGs and MAIs, including Nogo, have been considered to play a pivotal role in suppressing axonal regeneration after CNS injury and in promoting neurodegeneration, and inhibition of these signals can prevent neurodegeneration [29–33]. In ALS, distal axonal degeneration in the peripheral nervous system (PNS) is an early pathological feature [34]. Previous studies showed that overexpression of Nogo-A in skeletal muscle was associated with NMJ dysfunction in ALS patients, and that inhibition of Nogo-A–NgR1 binding by anti–Nogo-A antibodies improved distal axonal degeneration and NMJ function and slowed disease progression in ALS model mice [11–13, 16]. However, despite this efficacy in mouse models, treatment with anti–Nogo-A antibodies was not found to provide any significant benefits to ALS patients [35]. We considered that this therapeutic failure could be partly due to the fact that signaling pathways downstream of NgR1 were insufficiently inhibited in NMJ and CNS, because several ligands besides Nogo-A, including other MAIs and CSPGs, can bind to and activate NgR1 [2, 36, 37]. In addition, since anti–Nogo-A antibodies are not thought to penetrate the blood–brain barrier, the only therapeutic target for these antibodies is Nogo-A expressed in the PNS, including in muscles [35], and there may be no benefits involving Nogo-A in the CNS.

As suggested by a previous study demonstrating early upregulation of CSPGs in the lumbar spinal cord in an H46R mutant SOD1 transgenic rat model [14], we hypothesized that the increased expression of NgR1 or MAIs may contribute to enhanced NgR1 signaling in the CNS of SOD1 Tg mice. However, expression levels of NgR1, Nogo-A, MAG, and OMgp remained unchanged (Fig. 1c–j), even at 20 weeks when neurodegeneration was already apparent (Fig. 4a, b). By contrast, we found a progressive, lifelong decrease in LOTUS expression levels in the lumbar spinal cord of SOD1 Tg mice (Fig. 1a, b). Interestingly, a decrease in LOTUS expression was also found in the spinal cords of postmortem ALS patients despite preserved NgR1 expression (Fig. 2), implying that LOTUS-related pathogenesis in the CNS might be similar between model mice and patients.

LOTUS is an endogenous NgR1 antagonist; while it is potentially beneficial because it can block the binding of all MAIs and CSPGs to NgR1 [21–24], a reduction in its expression can be a major cause of strongly enhanced NgR1 signaling. The possible usefulness of LOTUS as a potent NgR1 antagonist and the deleterious pathological effects of decreasing the levels of its endogenous form prompted us to exogenously express LOTUS as a treatment for SOD1 Tg mice. This transgenic approach restored LOTUS expression in the CNS and PNS and improved motor function and survival in SOD1 Tg mice. Pathological evidence of this phenotypic improvement in the CNS and PNS manifested as a reduction in each of the following: motor neuronal death, glial proliferation, and NMJ degeneration. Thus, the therapeutic strategy in the current study is quite different from that in previous studies that used anti–Nogo-A antibodies, because it involved inhibiting the binding of all ligands to NgR1 and suppressing signaling downstream of NgR1 both in the CNS and PNS, even though MAIs were not upregulated in the CNS.

To confirm that LOTUS exerted its function through NgR1, we examined the expression of ROCK2, a ROCK isoform mainly expressed in neurons, as well as phosphorylated LIM kinase 1 (LIMK1) and cofilin, all of which are known downstream molecules of NgR1 signaling. NgR1 activates the small GTPase RhoA, which binds to and activates ROCK to act on neuronal effectors. ROCK phosphorylates LIMK1, and LIMK1 phosphorylation mediates cofilin phosphorylation. ROCK also activates slingshot phosphatase 1 (SSH1), which mediates cofilin dephosphorylation. This cofilin phosphorylation–dephosphorylation cycle may be required to properly regulate actin polymerization and thereby mediate neurite outgrowth [38]. A previous study indicated that Nogo-66, the extracellular domain of Nogo, and myelin fractions including Nogo, MAG, and Omgp, disrupted cofilin phosphorylation–dephosphorylation cycling mediated by LIMK1 and SSH1, resulting in inhibition of neurite outgrowth [38]. In the current study, although ROCK2 expression was not altered in LOTUS Tg/SOD1 Tg mice, phosphorylation levels of LIMK1 and cofilin were comparable to those in WT mice, in contrast to significantly reduced phosphorylation in SOD1 Tg mice (Fig. 6c–f). This suggests that abnormal LIMK1/cofilin phosphorylation dynamics caused by NgR1 signaling in SOD1 Tg mice were restored to WT levels by LOTUS overexpression. In addition, we demonstrated a LOTUS-induced reduction of ataxin-2 in SOD1 Tg mice (Fig. 6g, h), which is further evidence that LOTUS modulates the NgR1 signaling pathway. Ataxin-2 was recently reported to be decreased by knockdown or peptide inhibition of NgR1 in cell culture, as well as by knockout of NgR1 in mice [39]. In ALS model mice overexpressing human Tar DNA–binding protein 43 (TDP-43), a decrease in ataxin-2 markedly improved survival and motor function by suppressing TDP-43 aggregation [40], and this led to the launch of a clinical trial of *ATXN2*-targeted antisense oligonucleotide treatment in ALS patients (ClinicalTrials.gov: NCT04494256). On the other hand, since TDP-43 aggregation does not occur in SOD1 Tg mice, it is unclear whether the LOTUS-induced reduction of ataxin-2 is directly involved in the improved disease conditions of LOTUS Tg/SOD1 Tg mice. However, we clearly showed that LOTUS overexpression modified downstream molecules of NgR1, including ataxin-2, and exerted a beneficial effect on SOD1 Tg mice although it was difficult to show the direct NgR1 signal pathway. This has promising implications for the application of a LOTUS-targeting therapeutic strategy to ALS patients with TDP-43 aggregation, a context in which ataxin-2 reduction may have important therapeutic effects.

Our study also showed that LOTUS overexpression suppressed gliosis in the lumbar spinal cords of SOD1 Tg mice. A previous study reported that NgR1 was expressed not only on neurons, but also partly on microglia, and that blocking of NgR1 signaling reduced NgR1-mediated microglial activation and the subsequent release of proinflammatory factors [41]. In addition to its direct beneficial effect on neurons, LOTUS overexpression might suppress gliosis and the ensuing neuroinflammation by blocking glial NgR1, which could also promote neuronal survival. However, further investigations are needed to explore the effects of LOTUS on glial NgR1.

The signaling cascades of the neurotrophin BDNF serve many neuroprotective functions, including cell survival, axon and dendrite growth, and augmentation of synaptic plasticity through binding of BDNF to its high-affinity tyrosine kinase receptor, tropomyosin-related kinase B (TrkB) [42]. In this study, we observed that LOTUS overexpression upregulated BDNF, but not NGF or NT-3, in the lumbar spinal cords of SOD1 Tg mice (Fig. S2a). This finding is consistent with a recent study of mice in a spinal cord injury model in which successful transplantation therapy was achieved by LOTUS-expressing human induced pluripotent stem cell–derived neural stem/progenitor cells with upregulated BDNF [22].

Thus, LOTUS might have multiple neuroprotective effects on ALS motor neurons in both the CNS and PNS, including overproduction of BDNF, NgR1-blockade–mediated promotion of neurite outgrowth, and possible suppression of neuroinflammation via glial NgR1 blocking. Together these processes eventually prevent neurodegeneration and ameliorate disease phenotypes. The effects of LOTUS may be CNS-predominant in our therapeutic paradigm, considering that significantly improved survival of spinal motor neurons began at 20 weeks (Fig. 4a, b), whereas that of nerves innervating the NMJ occurred at 24 weeks (Fig. 5).

One limitation of this study is that there was insufficient prolongation of survival in LOTUS Tg/SOD1 Tg mice, although survival was significantly longer than in SOD1 Tg mice (Fig. 3c, d). Following LOTUS overexpression in SOD1 Tg mice, the proportion of phosphorylated cofilin recovered to the level seen in WT mice, but was not significantly different from that in SOD1 Tg mice (Fig. 6e, f). One possible reason for these observations is that the expression level of the LOTUS transgene (2.5-fold higher than endogenous level) (Fig. 3a, b), was not high enough to sufficiently reduce ALS pathogenesis and improve clinical phenotypes. Another limitation of this study is that the use of LOTUS Tg is not a clinically applicable approach for LOTUS overexpression. To overcome these limitations, we are beginning to employ viral vector injection to deliver therapeutically sufficient levels of LOTUS to the nervous system. Another possible treatment approach is to identify or develop small-molecule compounds to upregulate LOTUS expression, taking advantage of the fact that LOTUS is an endogenous NgR1 antagonist. Finally, although we showed that LOTUS overexpression restored LIMK1/cofilin phosphorylation dynamics, this is not direct evidence of reduced NgR1 signaling by LOTUS because there are other pathways that influence these dynamics. However, our previous study showed that among Nogo receptor family members, LOTUS directly bound specifically to NgR1 and exerted antagonistic effects [18]. We further demonstrated that ataxin-2, which was recently shown to be another downstream molecule of NgR1 signaling [39], was reduced by LOTUS overexpression. On the basis of these findings, we hypothesize that LOTUS may have reduced NgR1 signaling in this study.

In conclusion, we observed a significant decrease in LOTUS expression in both SOD1 Tg mice and postmortem ALS patients. We also confirmed that LOTUS overexpression in SOD1 Tg mice resulted in modestly but significantly improved motor function and lifespan extension, accompanied by pathological amelioration. Our results indicate that restoring LOTUS is a promising therapeutic strategy for ALS.

## Materials and methods

### Animals

All animal experiments were conducted under protocols approved by the Animal Experiment Committee of Yokohama City University (approval number: F-A-14-1). C57BL/6J (B6) mice were purchased from Japan SLC (Hamamatsu, Japan). LOTUS Tg mice were generated using the mouse synapsin-1 promoter, which overexpresses hemagglutinin (HA)-tagged LOTUS specifically in neurons, as previously described [20]. Transgenic mice carrying 23 copies of a transgene encoding the G93A mutant of human SOD1 (designated as SOD1 Tg mice) were purchased from the Jackson Laboratory (B6.Cg-Tg(SOD1-G93A)1Gur/J; #004435, Jackson Laboratory, Bar Harbor, ME, USA) [43]. LOTUS-overexpressing SOD1 Tg mice, which harbor a homozygous LOTUS transgene in SOD1 Tg mice (designated as LOTUS Tg/SOD1 Tg mice), were obtained by crossing SOD1 Tg mice with LOTUS Tg mice.

### Behavioral assessments

Female mice were randomly chosen, and body weights were measured weekly after 8 weeks. After 10 weeks, motor function was assessed weekly in a non-blinded manner using the Rotarod test (Ugo-Basile, Monvalle, Varese, Italy: constant speed, 15 rpm; cut-off time, 300 s), and the wire hang test was also performed weekly (cut-off time, 60 s) [44]. For the Rotarod test, each mouse was evaluated three times. For the wire hang test, each mouse was evaluated once.

### Immunoblotting analysis

Tissue samples were homogenized in lysis buffer (20 mM Tris-HCl, pH 8.0, 150 mM NaCl, 1% Nonidet P-40) supplemented with Complete Mini protease inhibitor cocktail (#11836153001; Sigma-Aldrich, St. Louis, MO, USA), 1% phosphatase inhibitor cocktail 2 (# P5726; Sigma-Aldrich), and 1% phosphatase inhibitor cocktail 3 (# P004; Sigma-Aldrich), and proteins were electrophoretically separated on 7.5% or 5–20% polyacrylamide gels and transferred onto polyvinylidene difluoride membranes. Membranes were blocked with 5% skim milk in Tris-buffered saline containing 0.1% Tween 20 (TBST) for 1 h at room temperature (RT) and then incubated overnight at 4 °C with the following primary antibodies: LOTUS (1:5,000; ITM, Matsumoto, Japan); NgR1 (1:2000, #AF1440; R&D Systems, Minneapolis, MN, USA); human NgR (1:10,000, #AF1208; R&D Systems); Nogo-A (1:1000, #13401; Cell Signaling Technology, Danvers, MA, USA); MAG (1:1000, #12275; Cell Signaling Technology); OMgp (1:20,000, #AF1674; R&D Systems); Rho-associated coiled-coil-containing protein kinase 2 (ROCK2) (1:100, #sc-398519; Santa Cruz Biotechnology, Dallas, TX, USA); p-cofilin (Ser3) (1:1000, #3313; Cell Signaling Technology); cofilin (1:1000, #5175; Cell Signaling Technology); p-LIMK1 (Thr508) (1:1000, #3841; Cell Signaling Technology); ataxin-2 (1:1000, #HPA018295; Atlas Antibodies); and β-actin (1:10,000, #A5441; Sigma-Aldrich). After the membranes were washed with 1% skim milk in TBST, they were incubated with horseradish peroxidase-conjugated secondary antibodies for 1 h at RT. The bands were visualized using an enhanced chemiluminescence reagent (Immobilon^R^ Forte Western HRP Substrate, #WBLUF0100; Millipore, Burlington, MA, USA; or ECL Select^TM^ Western Blotting Detection Reagent, #RPN2235; Cytiva, Tokyo, Japan) and an ImageQuant LAS 4000 instrument (GE Healthcare, Chicago, IL, USA). The quantification of each band was performed using ImageJ software (https://imagej.nih.gov/ij/) [45]. In addition, the full and uncropped western blots were uploaded as “Supplementary Material”.

### Histological analysis

SOD1 Tg mice and LOTUS Tg/SOD1 Tg mice were anesthetized with 0.3 mg/kg of medetomidine, 4.0 mg/kg of midazolam, and 5.0 mg/kg of butorphanol and euthanized at 20 and 24 weeks [46]. Mice were transcardially perfused with phosphate-buffered saline (PBS) followed by 4% paraformaldehyde (PFA) in PBS. Lumbar spinal cords were dissected, and tissues were immediately fixed in 4% PFA and embedded in paraffin. Blocked spinal cords were cut into 6-μm cross-sections. Motor neuron loss and gliosis in the ventral horn of the lumbar spinal cord were evaluated in three to five sections per mouse (SOD1 Tg: n = 5; LOTUS Tg/SOD1 Tg: n = 5). For the quantitative analysis of motor neurons, staining was performed using cresyl violet, then the number of large cells (≥ 200 μm^2^) in each hemisection of the ventral horn of the lumbar spinal cord was counted [47]. For immunofluorescence staining of glial cells, paraffin-embedded sections were permeabilized with 0.1% Triton X-100 in PBS for 10 min, blocked with 5% normal goat serum for 60 min, and incubated overnight with rat anti–rabbit Iba1 polyclonal antibody (1:500, #019-19741; FUJIFILM Wako, Osaka, Japan) and anti–mouse glial fibrillary acidic protein (GFAP) monoclonal antibody (1:1000, #G3893; Sigma-Aldrich). Sections were subsequently incubated with secondary antibody conjugated with Alexa Fluor 488 (1:1000, #A-11034; Thermo Fisher Scientific, Waltham, MA, USA). Gliosis in the ventral horn of the lumbar spinal cord was evaluated by dividing the area immunostained for GFAP or Iba-1 by the ventral horn area using a deconvolution fluorescence microscopy system (BZ-X800; Keyence, Osaka, Japan).

### Neuromuscular junction (NMJ) analysis

Tibialis anterior (TA) muscles were dissected and fixed with 2% PFA in PBS for 10 min at RT. Fixation was followed by cryoprotection with 20% sucrose in PBS overnight. TA muscles were frozen in Tissue-Tek^®^ O.C.T. Compound (Sakura Finetek, Tokyo, Japan), and 40-mm-thick sections were made using a cryostat (Tissue-Tek^®^ Cryo3^®^; Sakura Finetek). Four sections per animal were collected and placed on glass slides. Muscle sections were stained with mouse anti–synaptic vesicle protein 2 (SV2) (1:100, #SV2; Developmental Studies Hybridoma Bank, Iowa City, IA, USA) and mouse anti-neurofilament (NF) (1:10,000, #2H3; Developmental Studies Hybridoma Bank) for 48 h at 4°C. Alexa Fluor 594–labeled α-bungarotoxin (α-BTX; 1:500, #B-13423; Thermo Fisher Scientific) and mouse Alexa Fluor 488 (1:500, #A-11017; Thermo Fisher Scientific) were subsequently added to the samples, followed by overnight incubation at 4°C. Images were obtained with a deconvolution fluorescence microscope system (BZ-X800; Keyence). Colocalization of NF and α-BTX was verified by creating z-stack images at 20× magnification.

The percentage of innervated NMJs was measured at 42–164 randomly selected synaptic sites per mouse (SOD1 Tg: n = 5; LOTUS Tg/SOD1 Tg: n = 6). Endplate occupancy was determined by assessing the extent of overlap of axon terminal signals (labeled by antibodies against SV2 and NF) with endplate signals (labeled by α-BTX). Endplates were scored as “fully denervated” when no endplates were deemed to be occupied by an axon terminal. By contrast, “fully innervated” indicates 100% occupancy, “fully innervated” indicates no occupancy, and “partially innervated” indicates occupancy values between 0% and 100%.

### Human samples

The spinal cords of four patients with sporadic ALS (SALS) and four control patients with non-ALS diseases (epilepsy, argyrophilic grain dementia, Parkinson’s disease, and myasthenia gravis) who underwent autopsies from 2016 to 2021 at Yokohama City University Hospital were analyzed. The ventral horn of each cryopreserved lumbar spinal cord was carefully dissected with the naked eye using the gray matter as a landmark, and then analyzed by immunoblotting. This study was approved by the institutional review board of Yokohama City University School of Medicine (approval number: B140109013). Informed consent was obtained from all individuals included in this study. The following information from medical records and clinical summaries was reviewed: age at death, sex, diagnosis, and disease duration. Clinical features of SALS and control cases are summarized in Table S2.

### Statistical analysis

Statistical significance was analyzed using Student’s *t*-test or one-way analysis of variance (ANOVA) followed by post hoc Tukey’s test. Survival time was analyzed by the Gehan–Breslow–Wilcoxon test. All statistical analyses were performed using GraphPad Prism version 9 (Graph Pad Software, La Jolla, CA, USA).

### Supplementary information

supplemental figure

Supplementary Materials and Methods

supplemental figure legends

supplemental table

Uncropped WB

## Data Availability

The datasets analyzed during the current study are available from the corresponding authors upon reasonable request.
