# Supplementary figures and images for: Lateral olfactory tract usher substance (LOTUS), an endogenous Nogo receptor antagonist, ameliorates disease progression in amyotrophic lateral sclerosis model mice
Source: Cell Death Discov. 2023 Dec 14;9:454. doi: 10.1038/s41420-023-01758-7 (PMC10721829; doi:10.1038/s41420-023-01758-7)

Supplemental Figure S1

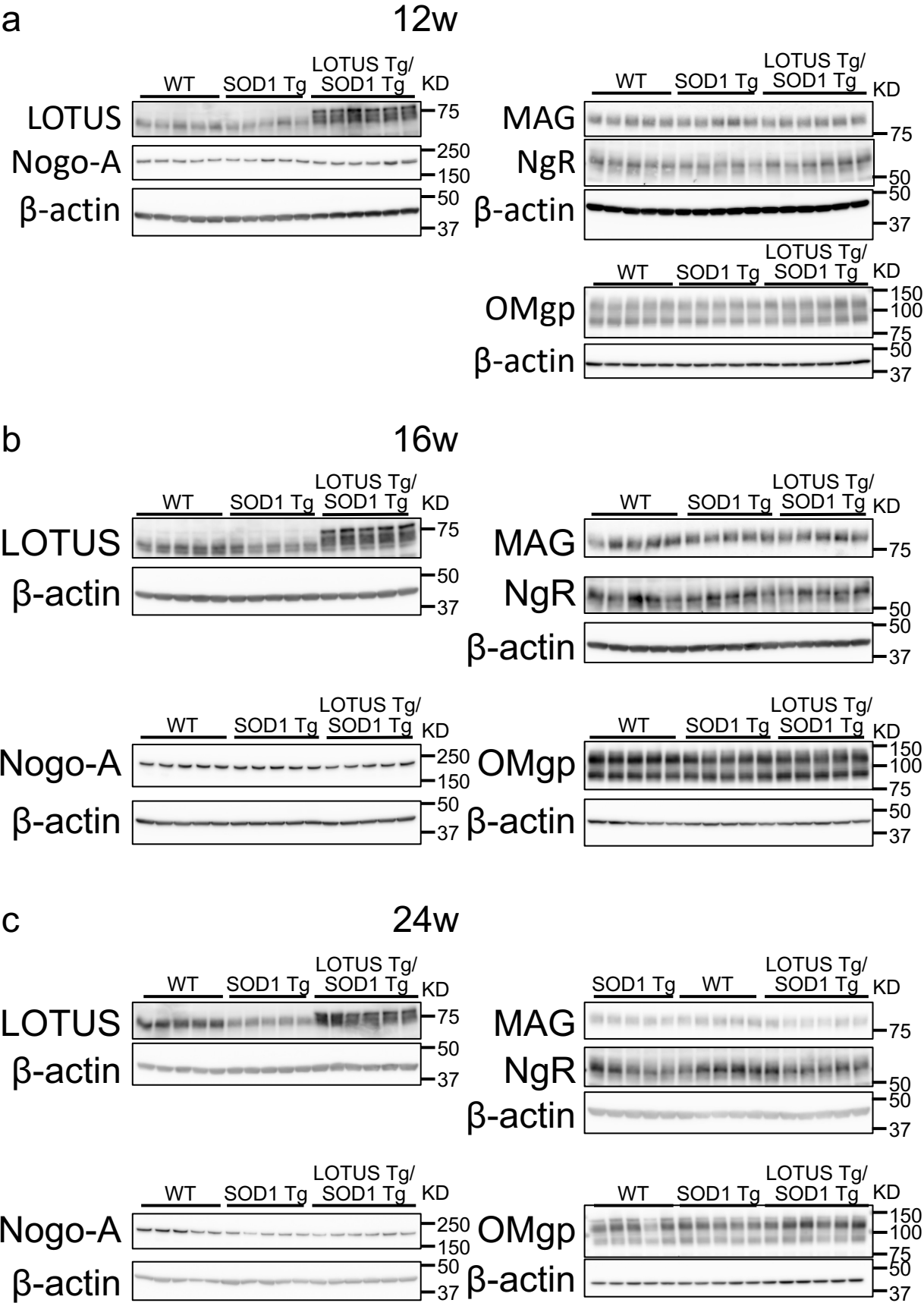

Supplemental Figure S2

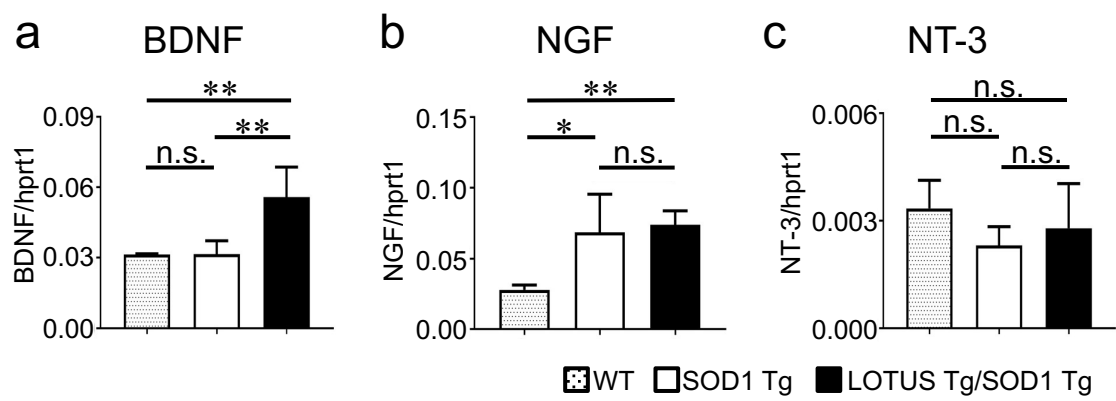

Supplement: Supplementary file 1 — supplemental figure [file 41420_2023_1758_MOESM1_ESM.pdf]
