# Supplementary material for: Lateral olfactory tract usher substance (LOTUS), an endogenous Nogo receptor antagonist, ameliorates disease progression in amyotrophic lateral sclerosis model mice
Source: Cell Death Discov. 2023 Dec 14;9:454. doi: 10.1038/s41420-023-01758-7 (PMC10721829; doi:10.1038/s41420-023-01758-7)
Supplement: Supplementary file 2 — Supplementary Materials and Methods [file 41420_2023_1758_MOESM2_ESM.docx]

**Lateral olfactory tract usher substance (LOTUS), an endogenous Nogo receptor antagonist, ameliorates disease progression in amyotrophic lateral sclerosis model mice**

Takuya Ikeda^1^, Keita Takahashi^1^*, Minatsu Higashi^1^, Hiroyasu Komiya^1^, Tetsuya Asano^1^, Akihiro Ogasawara^1^, Shun Kubota^1^, Shunta Hashiguchi^1^, Misako Kunii^1^, Kenichi Tanaka^1^, Mikiko Tada^1^, Hiroshi Doi^1^, Hideyuki Takeuchi^1^, Kohtaro Takei^2^, Fumiaki Tanaka^1^*

1. Department of Neurology and Stroke Medicine, Yokohama City University Graduate School of Medicine, Yokohama, 236-0004, Japan
2. Molecular Medical Bioscience Laboratory, Yokohama City University Graduate School of Medical Life Science, Yokohama, 236-0004, Japan

*Corresponding author

**Supplementary Materials and methods**

**Quantitative reverse transcription PCR (qPCR) analysis**

Lumbar spinal cords were collected from 24-week-old mice, and total RNA was extracted using the miRNeasy Mini Kit (Qiagen, Valencia, CA, USA). For qPCR experiments, RNA was reverse transcribed into cDNA using SuperScript III (Life Technologies, Carlsbad, CA, USA). Levels of mRNAs encoding nerve growth factor (NGF), brain-derived neurotrophic factor (BDNF), neurotrophin-3 (NT-3), and hypoxanthine phosphoribosyl transferase 1 (HPRT1) were measured using qPCR, which was performed on a LightCycler using the KAPA SYBR FAST qPCR Master Mix Kit (Sigma-Aldrich, St. Louis, MO, USA). Relative expression levels were determined using the ΔΔCT method; the genes of interest were normalized against the geometric mean of HPRT1. Primers for qPCR are indicated in Supplemental Table S1.
