# Supplementary material for: Lateral olfactory tract usher substance (LOTUS), an endogenous Nogo receptor antagonist, ameliorates disease progression in amyotrophic lateral sclerosis model mice
Source: Cell Death Discov. 2023 Dec 14;9:454. doi: 10.1038/s41420-023-01758-7 (PMC10721829; doi:10.1038/s41420-023-01758-7)
Supplement: Supplementary file 3 — supplemental figure legends [file 41420_2023_1758_MOESM3_ESM.docx]

**Supplemental Figure legends**

**Figure S1. Immunoblotting analyses of LOTUS, NgR1, and MAIs in the lumbar spinal cords of WT, SOD1 Tg, and LOTUS Tg / SOD1 Tg mice at 12, 16, 24 weeks.**

(a) Immunoblots of LOTUS, Nogo-A, MAG, NgR1 and OMgp in the lumbar spinal cords at 12 weeks. (b) Immunoblots of LOTUS, Nogo-A, MAG, NgR1 and OMgp at 16 weeks. (c) Immunoblots of LOTUS, Nogo-A, MAG, NgR1 and OMgp at 24 weeks.

**Figure S2. Quantitative analyses of neurotrophic factors in lumbar spinal cords of WT, SOD1 Tg, and LOTUS Tg / SOD1 Tg mice.**

(a–c) qPCR data for neurotrophic factors in lumbar spinal cords at 24 weeks: (a) NGF, (b) BDNF, (c) NT-3. Values are means ± SD (n = 4). *, *p* < 0.05, **, *p* < 0.01 (one-way ANOVA followed by post hoc Tukey’s test).
