# Supplementary material for: Lateral olfactory tract usher substance (LOTUS), an endogenous Nogo receptor antagonist, ameliorates disease progression in amyotrophic lateral sclerosis model mice
Source: Cell Death Discov. 2023 Dec 14;9:454. doi: 10.1038/s41420-023-01758-7 (PMC10721829; doi:10.1038/s41420-023-01758-7)
Supplement: Supplementary file 4 — supplemental table [file 41420_2023_1758_MOESM4_ESM.docx]

**Supplemental Table S1. Primers for qPCR**

| Gene | Primer sequence |
| --- | --- |
| mouse NGF sense | GCAGTGAGGTGCATAGCGTA |
| mouse NGF antisense | CTGTGTCAAGGGAATGCTGA |
| mouse BDNF sense | GATGCCGCAAACATGTCTATGA |
| mouse BDNF antisense | TAATACTGTCACACACGCTCAGCTC |
| mouse NT-3 sense | GGAFTTTGCCGGAAGACTCTC |
| mouse NT-3 antisense | GGGTGCTCTGGTAATTTTCCTTA |
| mouse Hprt1 sense | TCAGTCAACGGGGGACATAAA |
| mouse Hprt1 antisense | CTGGTTAAGCAGTACAGCCCC |

**Supplemental Table S2. Characteristics of postmortem patients**

|  | Sex | Age | Diagnosis | Disease  duration | Cause of death |
| --- | --- | --- | --- | --- | --- |
| ALS1 | M | 57 y | sALS | 10 mo | Aspiration pneumonia |
| ALS2 | F | 84 y | sALS | 32 mo | Respiratory muscle paralysis |
| ALS3 | M | 74 y | sALS | 47 mo | Respiratory muscle paralysis |
| ALS4 | F | 71 y | sALS | 32 mo | Choking |
| ALS5 | M | 54 y | sALS | 25 mo | Respiratory muscle paralysis |
| ALS6 | M | 64 y | sALS | 24 mo | Respiratory muscle paralysis |
| ALS7 | M | 75 y | sALS | 5 mo | Respiratory muscle paralysis |
| CTL1 | F | 77 y | Epilepsy | - | Sudden death |
| CTL2 | F | 83 y | AGD | - | Urinary tract infection |
| CTL3 | F | 82 y | MG | - | Acute heart failure |

ALS, amyotrophic lateral sclerosis; CTL, control; M, male; F, female; y, years; sALS, sporadic ALS; AGD, argyrophilic grain dementia; MG, myasthenia gravis; mo, months
