# Supplementary material for: Lateral olfactory tract usher substance (LOTUS), an endogenous Nogo receptor antagonist, ameliorates disease progression in amyotrophic lateral sclerosis model mice
Source: Cell Death Discov. 2023 Dec 14;9:454. doi: 10.1038/s41420-023-01758-7 (PMC10721829; doi:10.1038/s41420-023-01758-7)

Full and uncropped western blot for Figure 1a
LOTUS

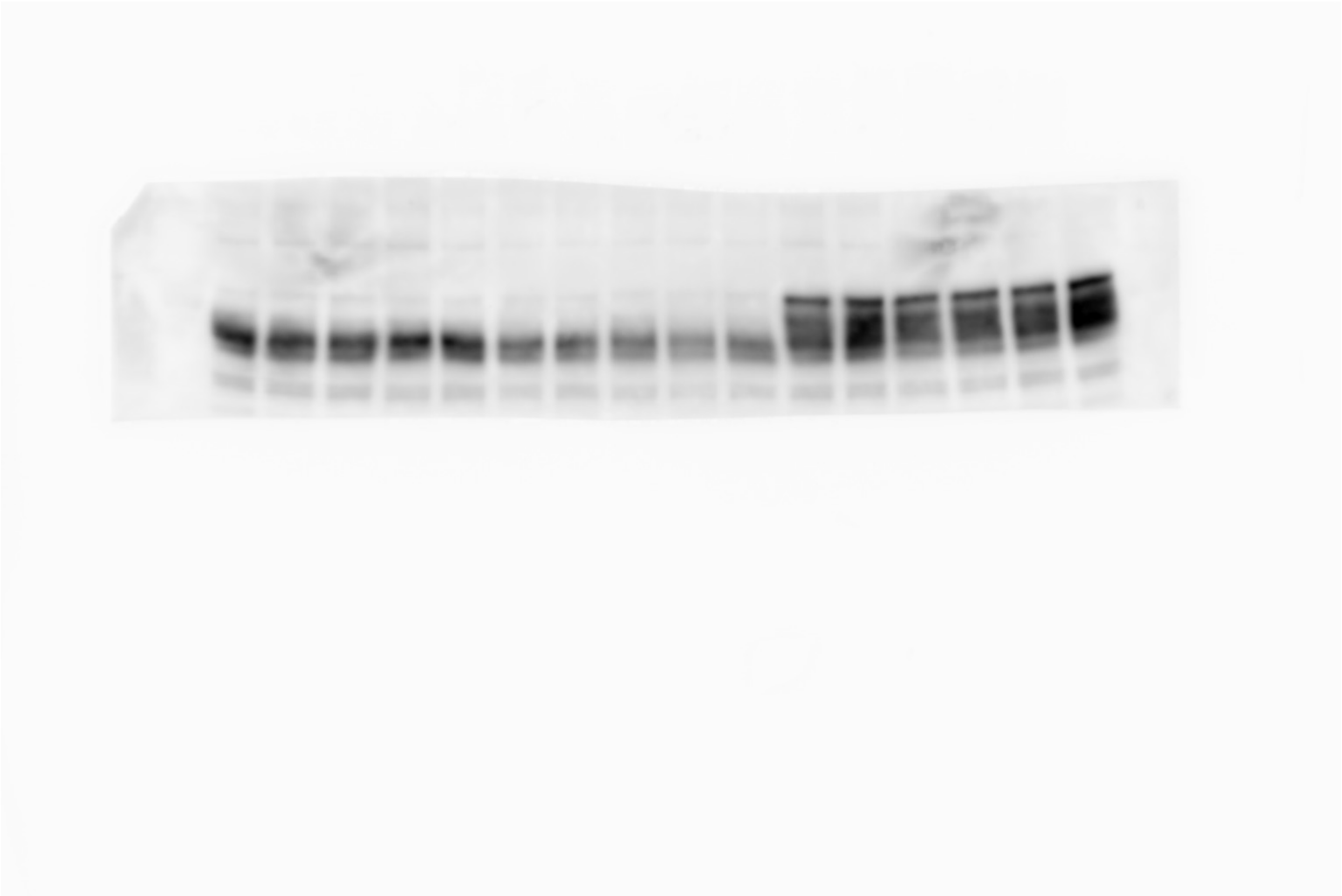

Full and uncropped western blot for Figure 1a
β-actin

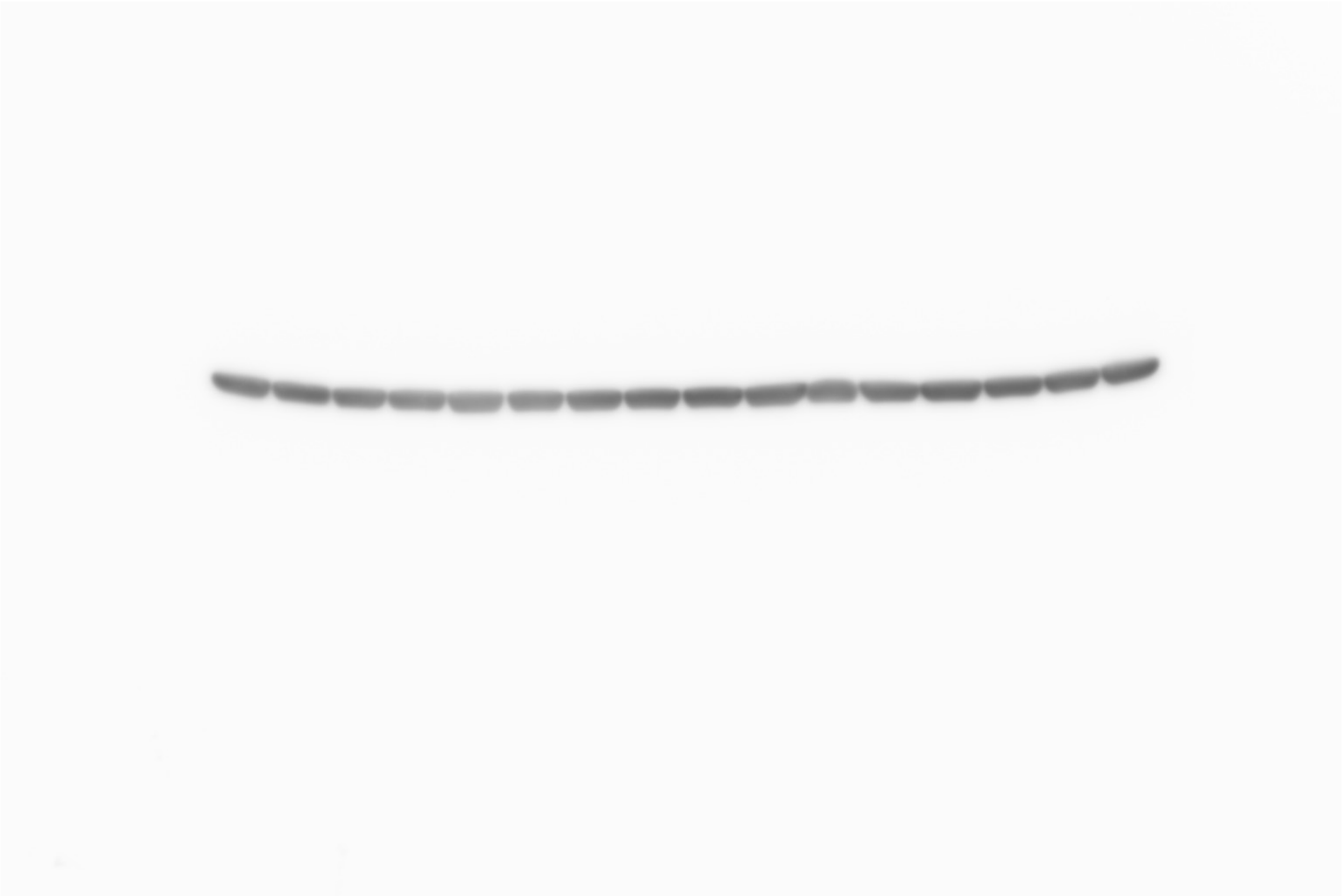

Full and uncropped western blot for Figure 1c
NgR1

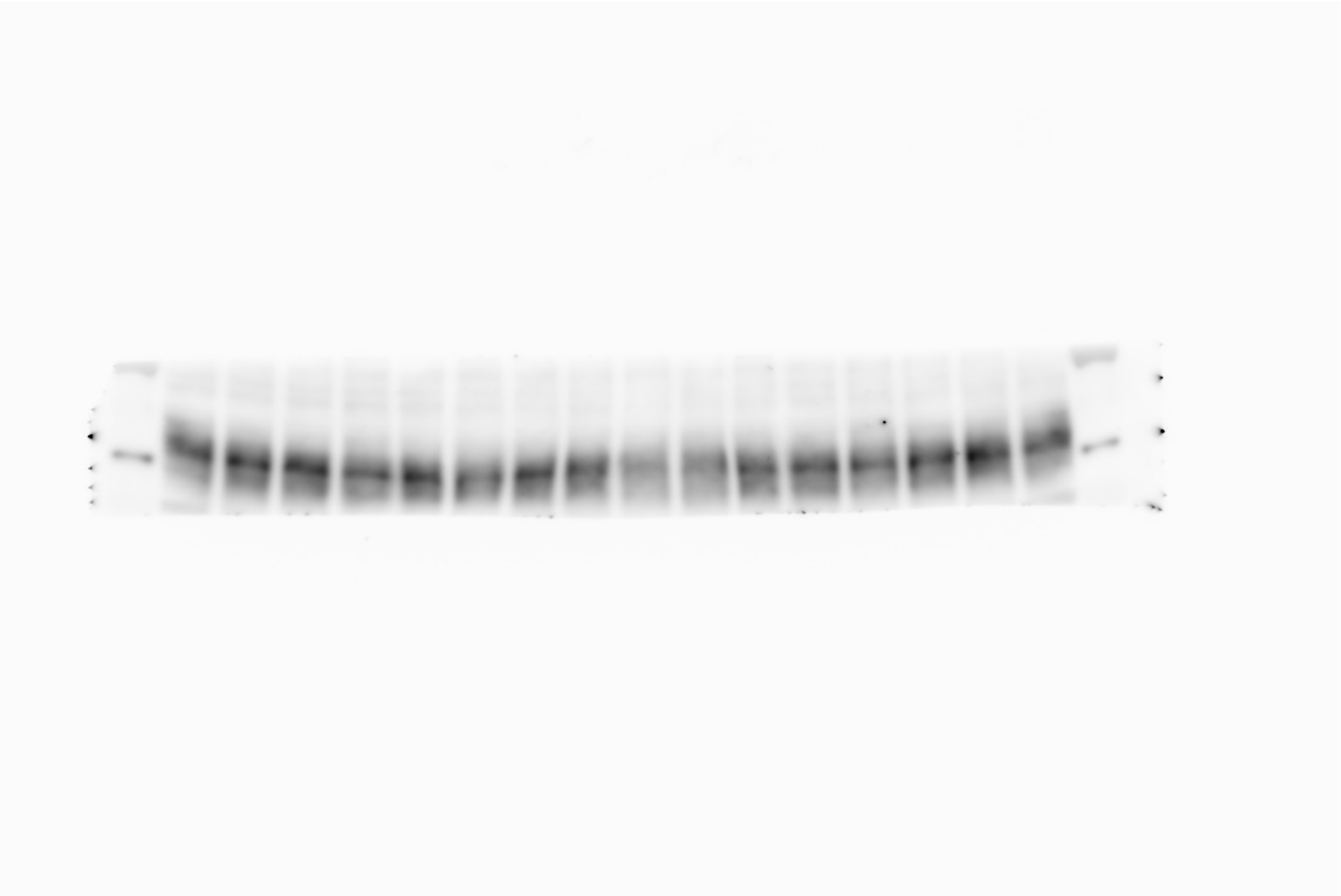

Full and uncropped western blot for Figure 1c
β-actin

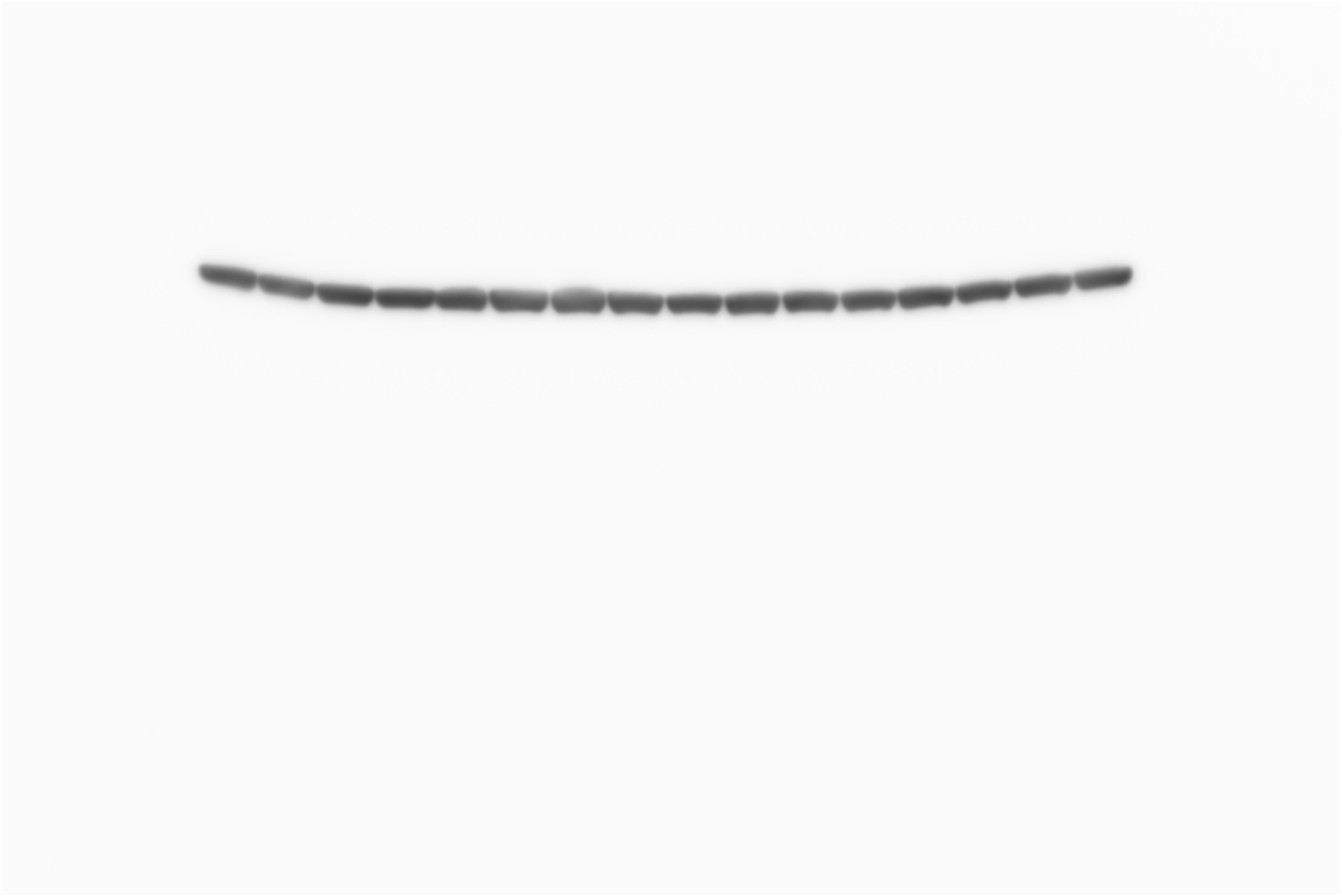

Full and uncropped western blot for Figure 1e
Nogo-A

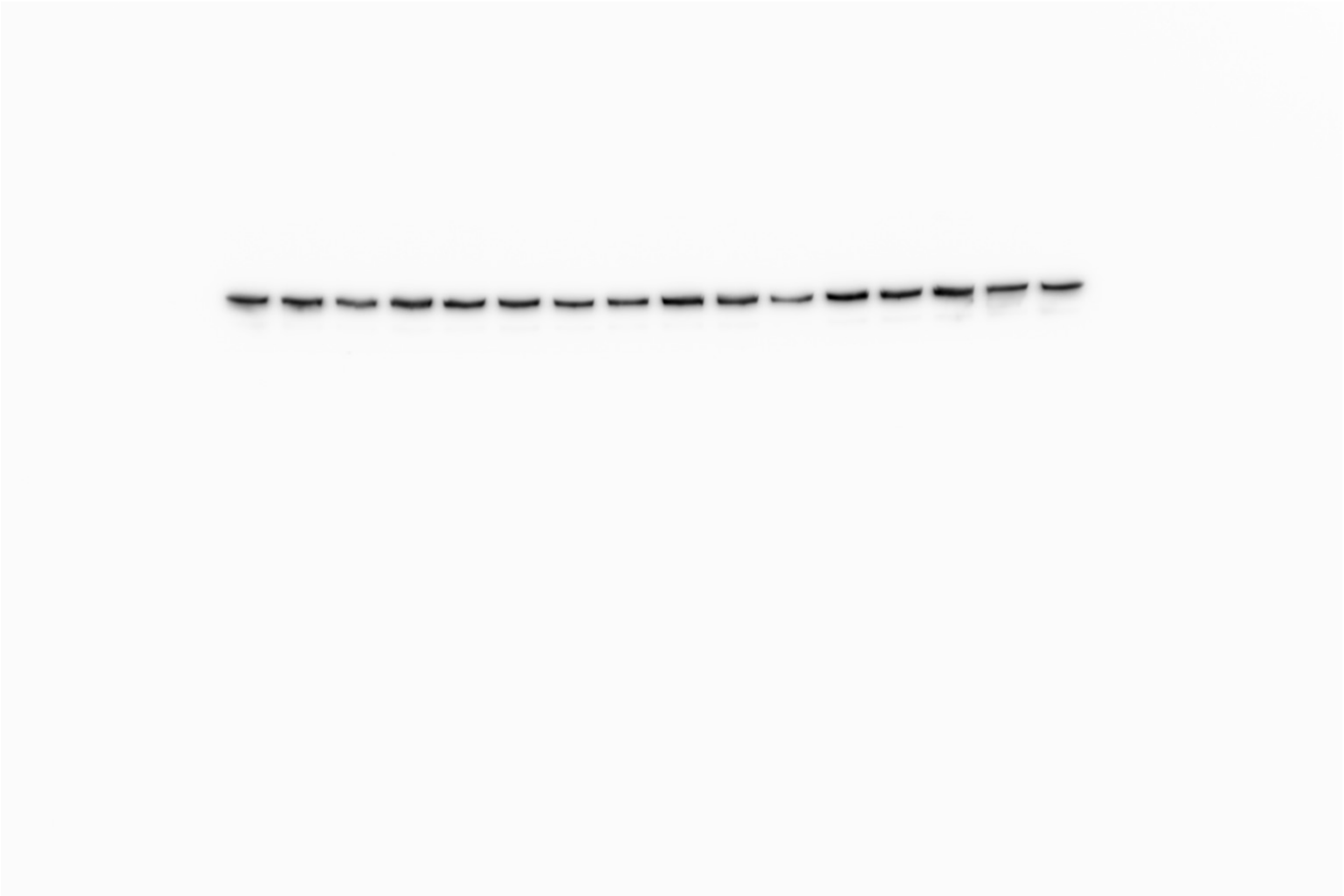

Full and uncropped western blot for Figure 1e
β-actin

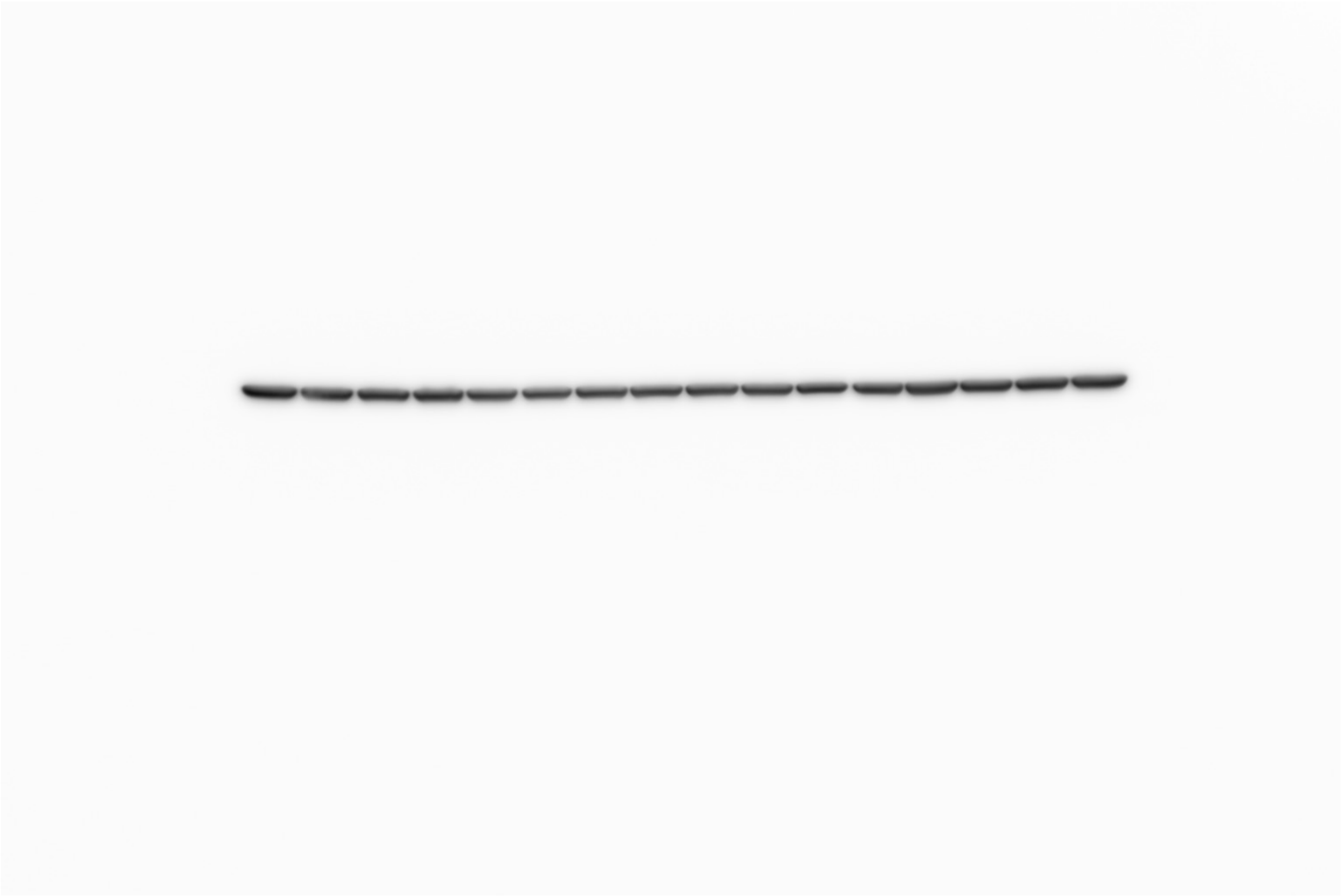

Full and uncropped western blot for Figure 1g
MAG

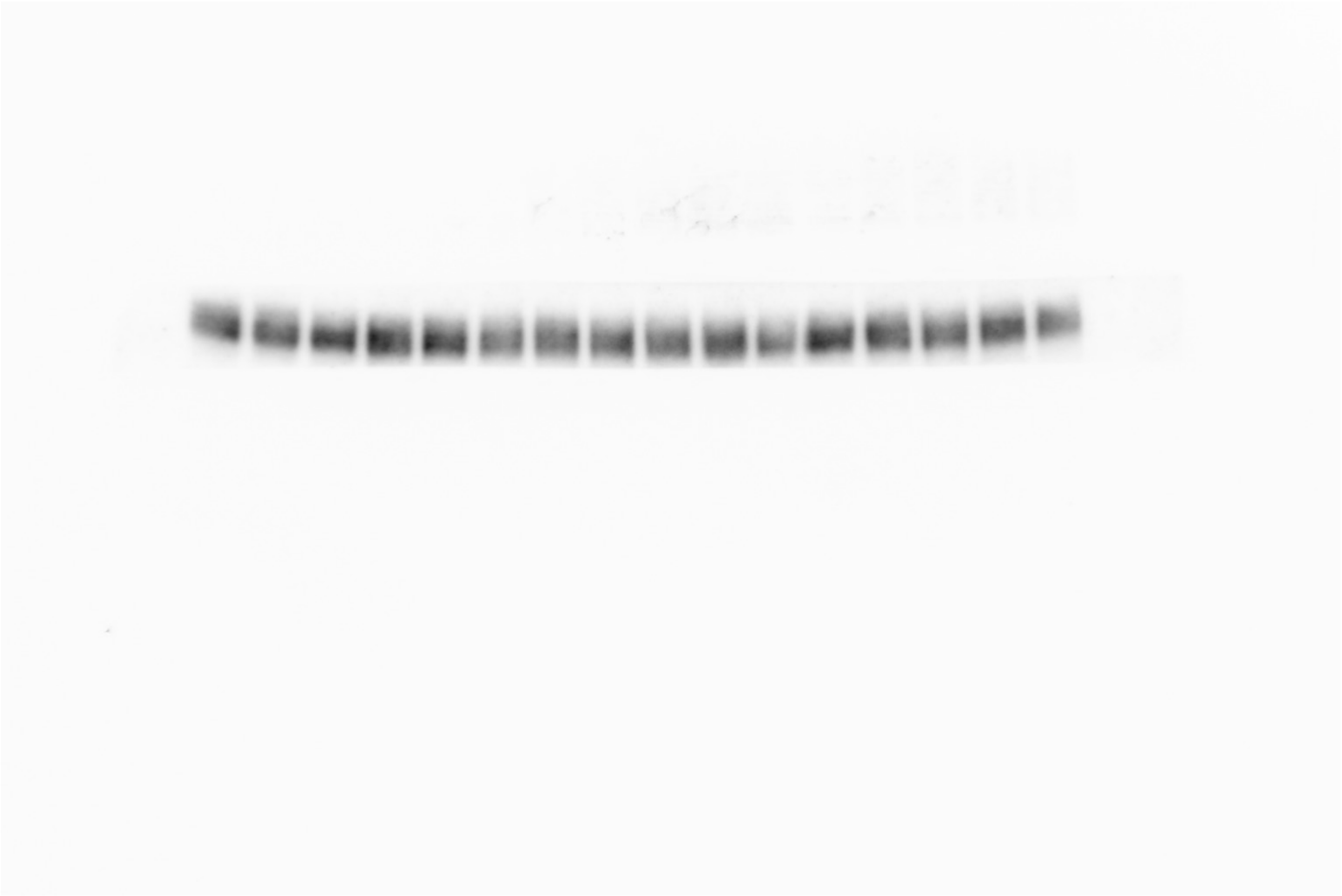

Full and uncropped western blot for Figure 1g
β-actin

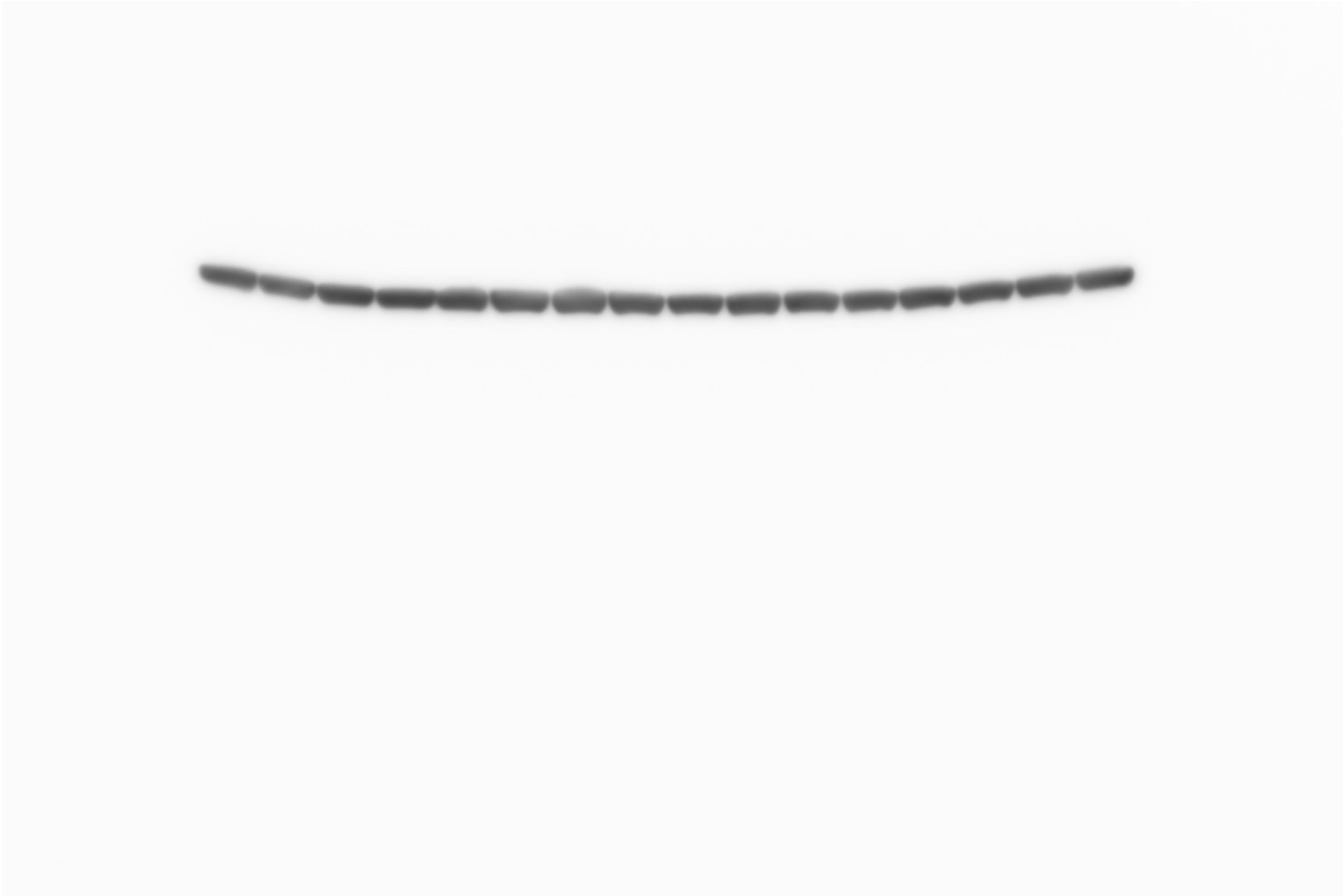

Full and uncropped western blot for Figure 1i
OMgp

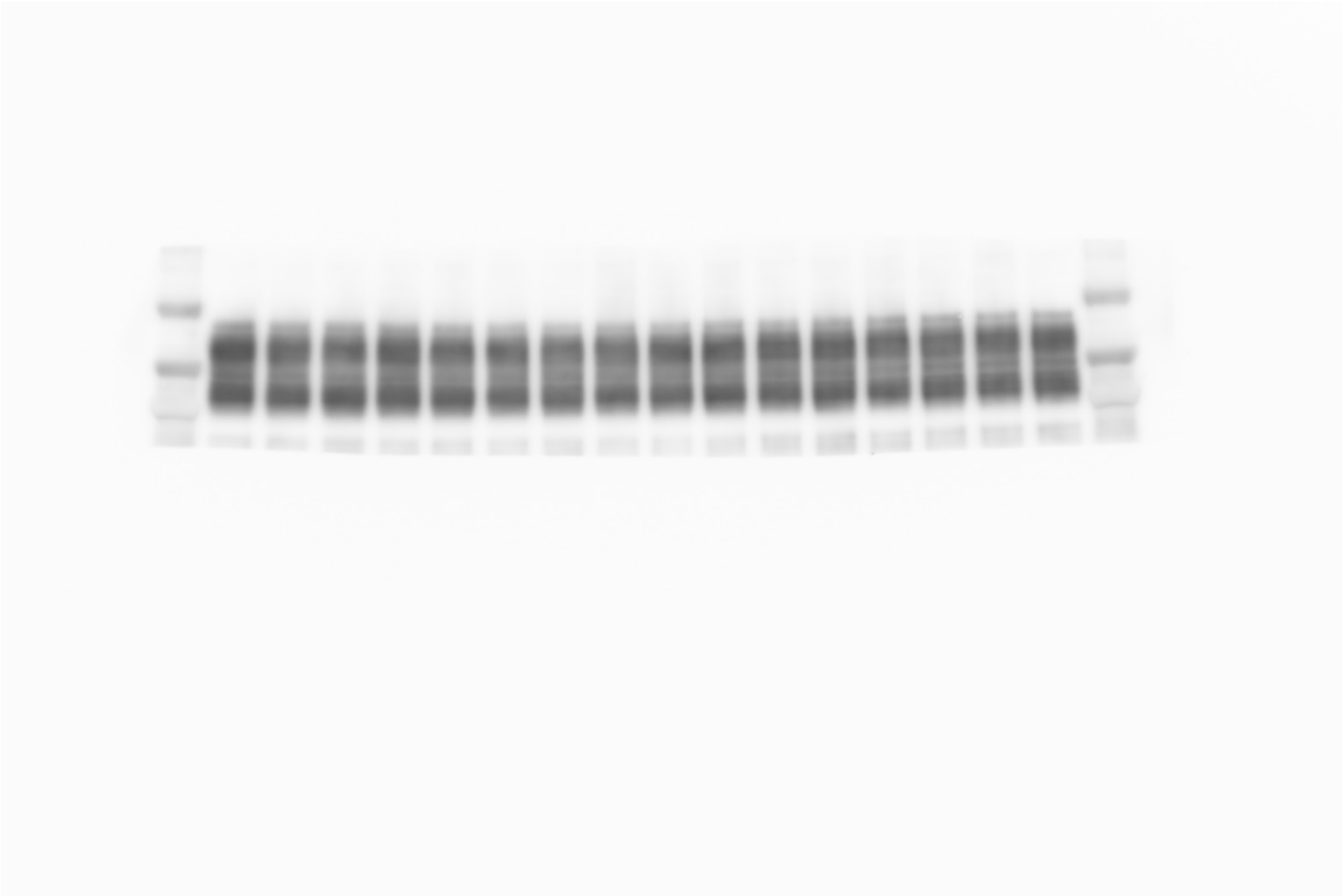

Full and uncropped western blot for Figure 1i
β-actin

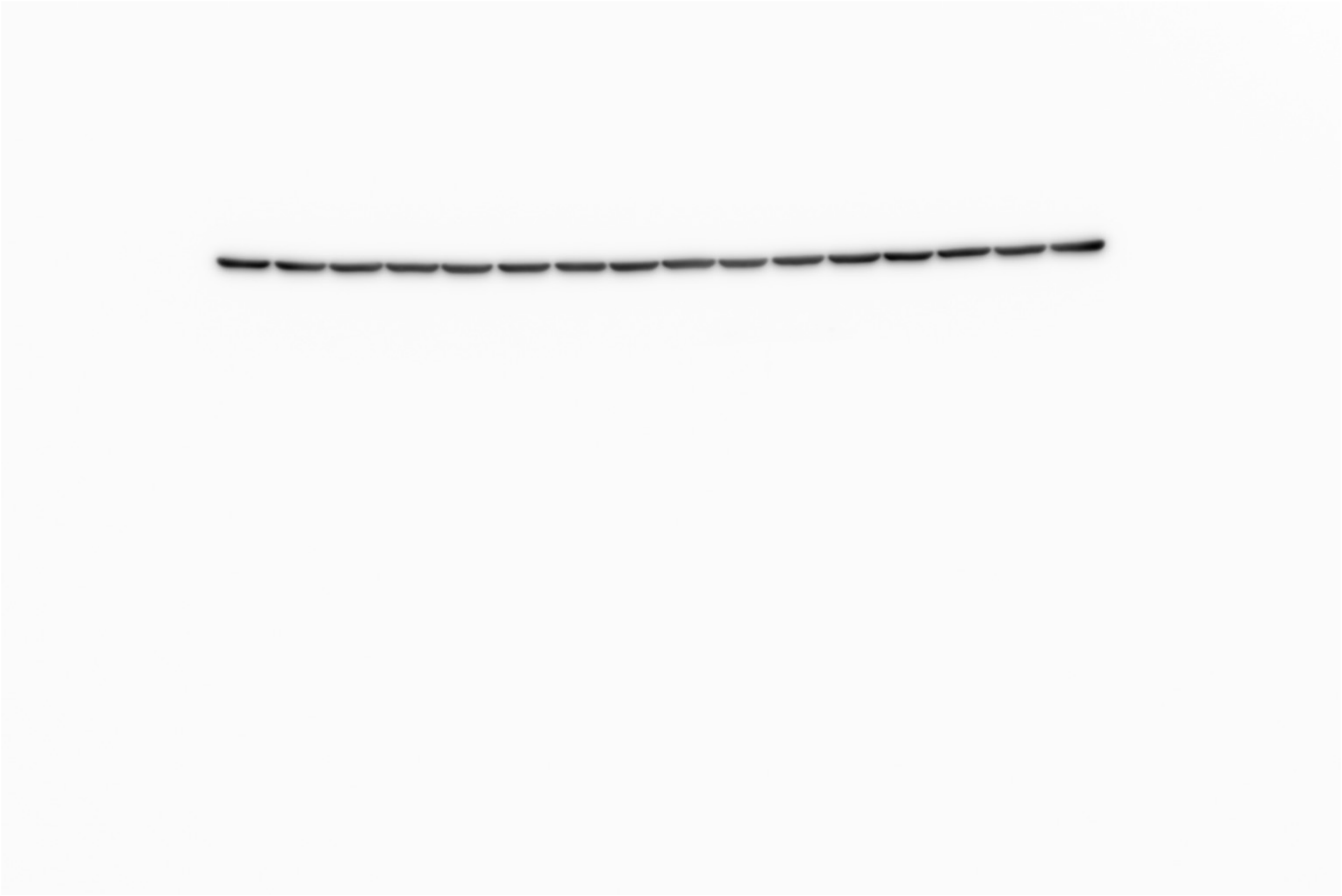

Full and uncropped western blot for Figure 2a
LOTUS

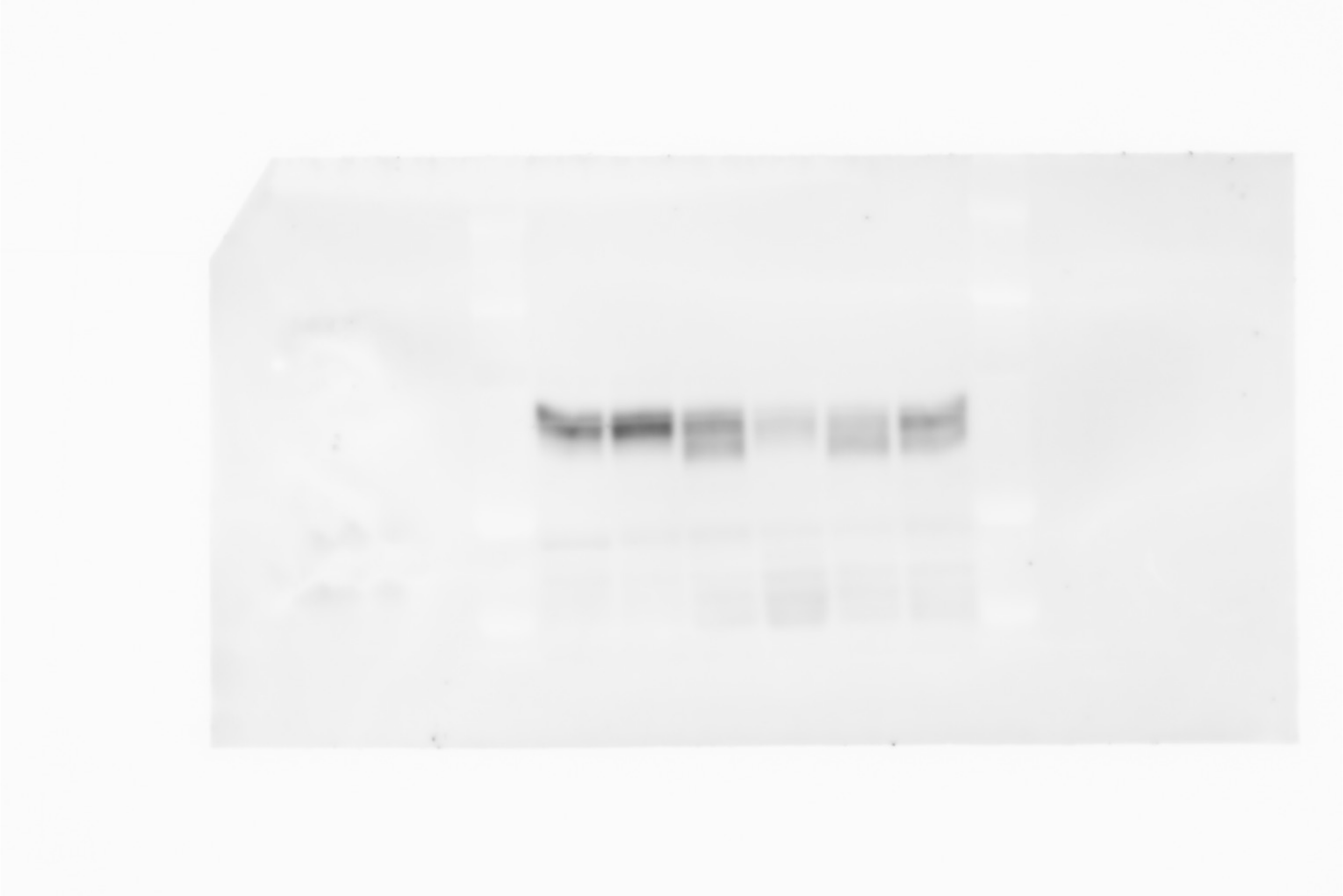

Full and uncropped western blot for Figure 2a
NgR

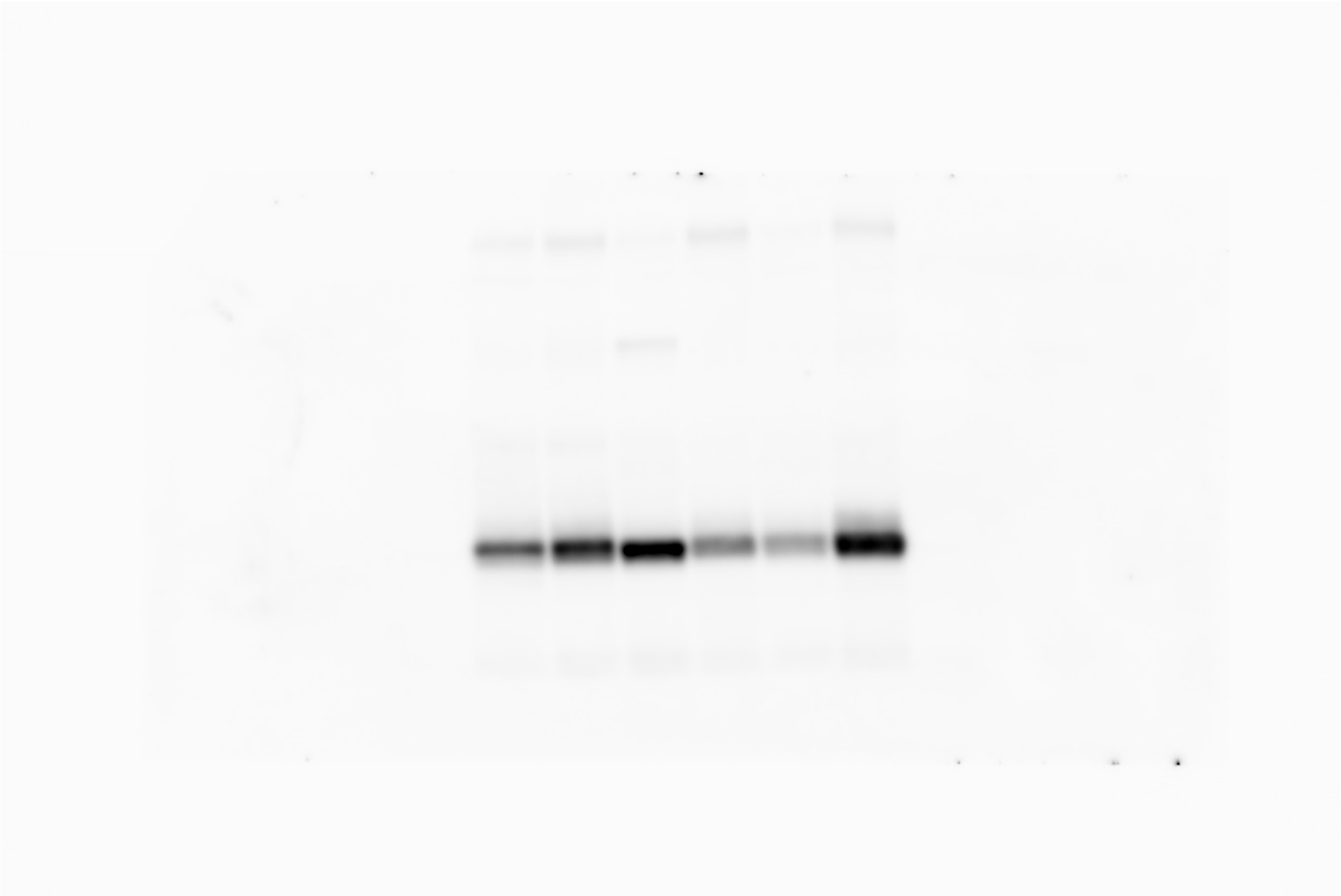

Full and uncropped western blot for Figure 2a
β-actin

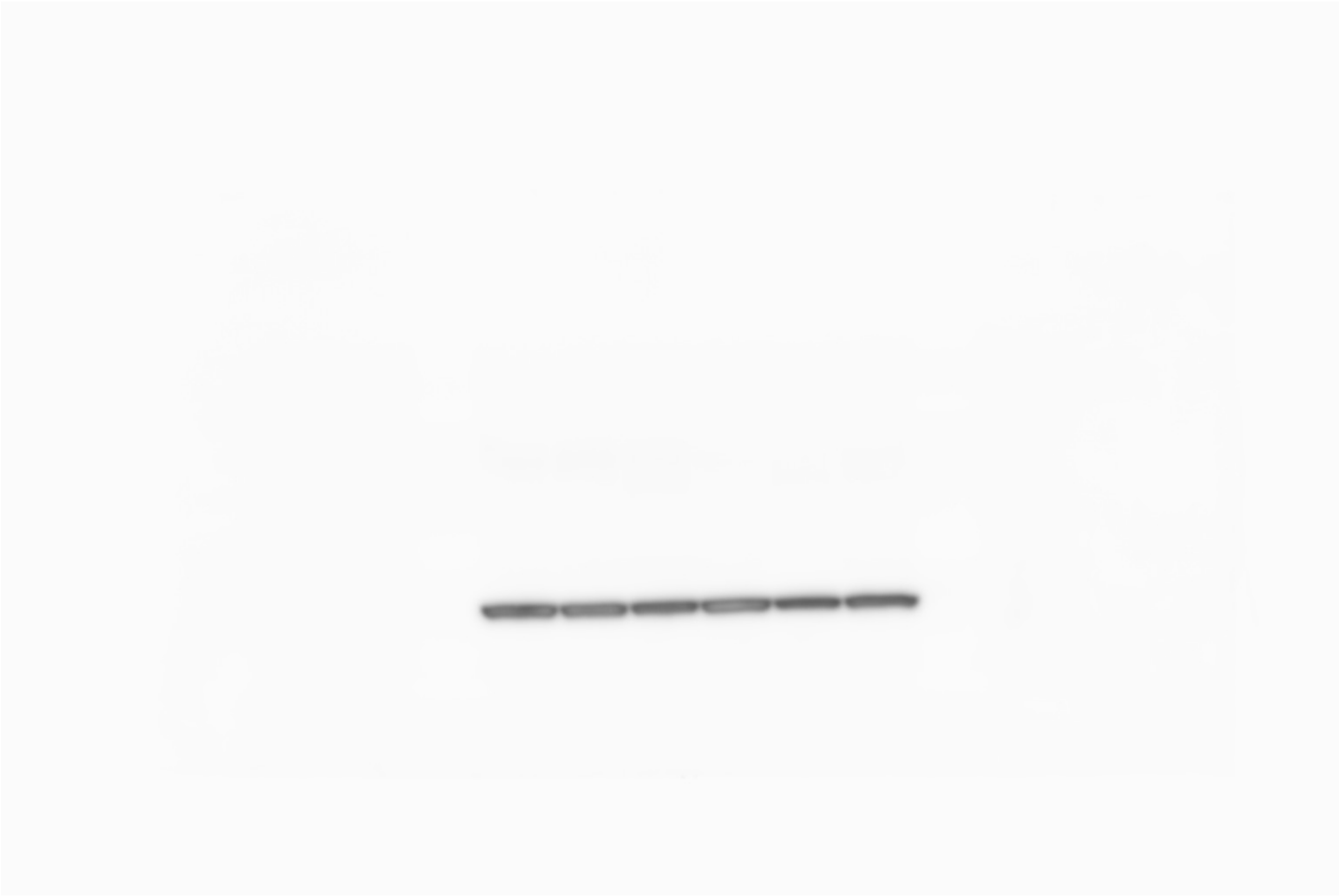

Full and uncropped western blot for Figure 3a
12w HA-LOTUS and LOTUS

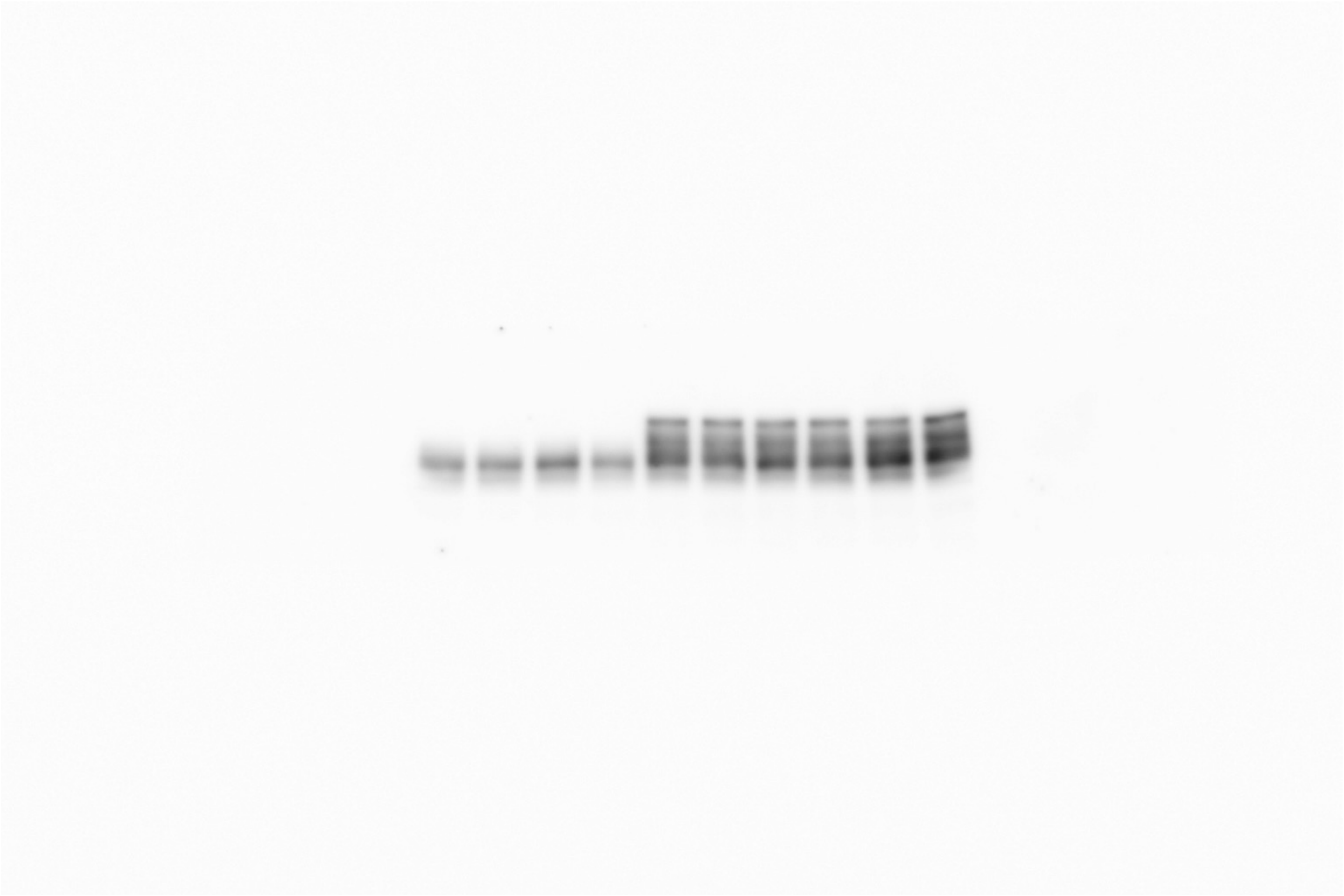

Full and uncropped western blot for Figure 3a
12w β-actin

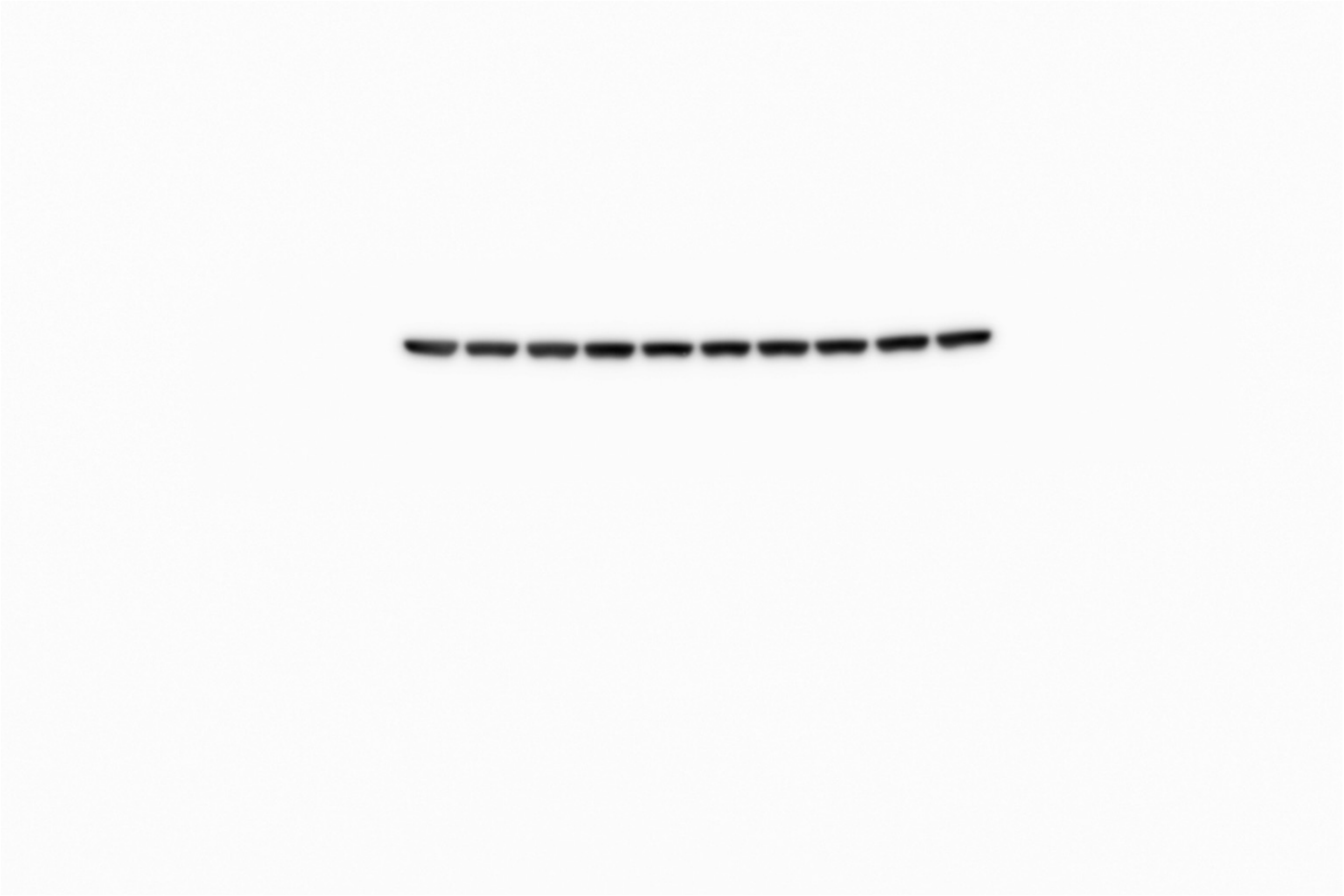

Full and uncropped western blot for Figure 3a
16w HA-LOTUS and LOTUS

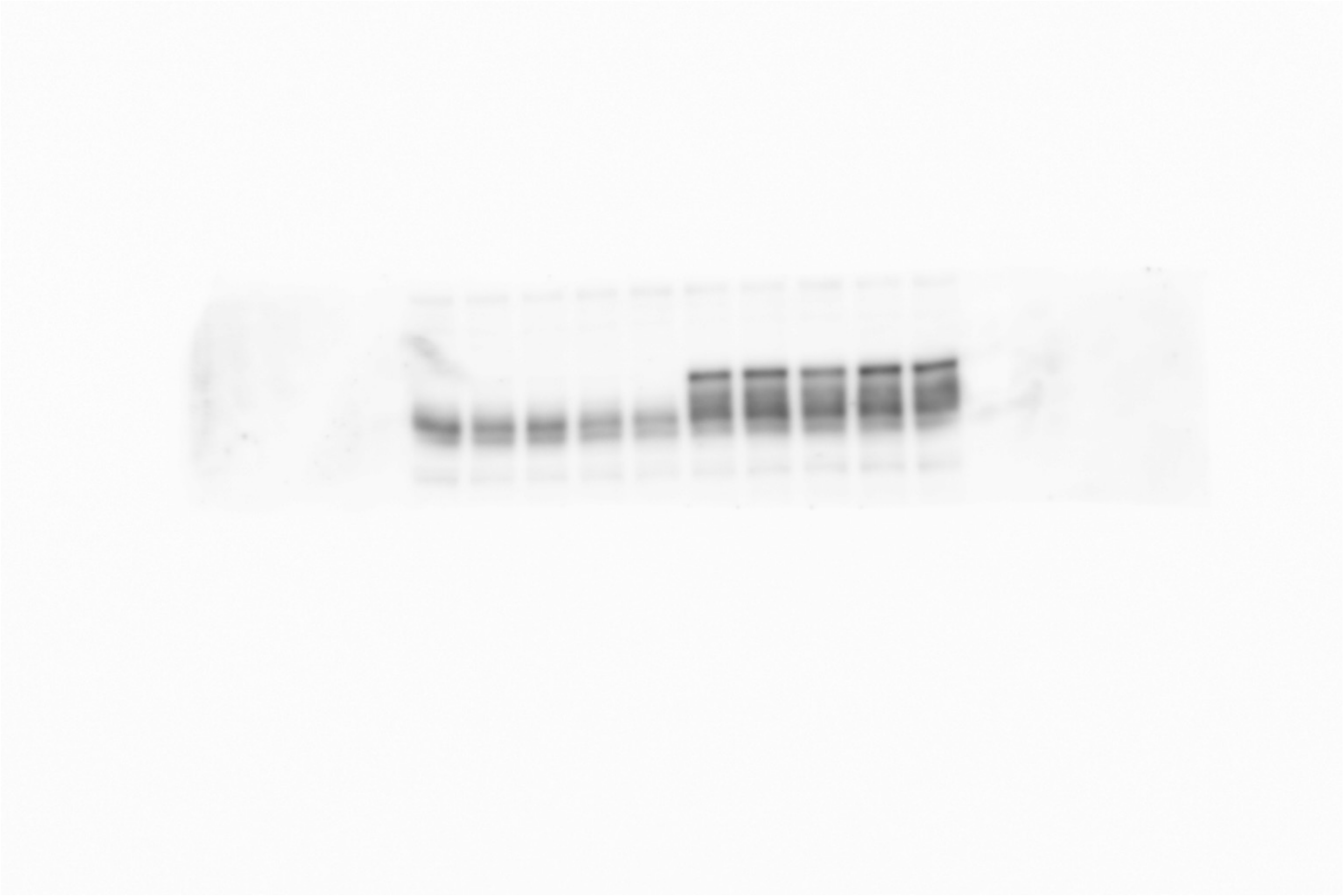

Full and uncropped western blot for Figure 3a
16w β-actin

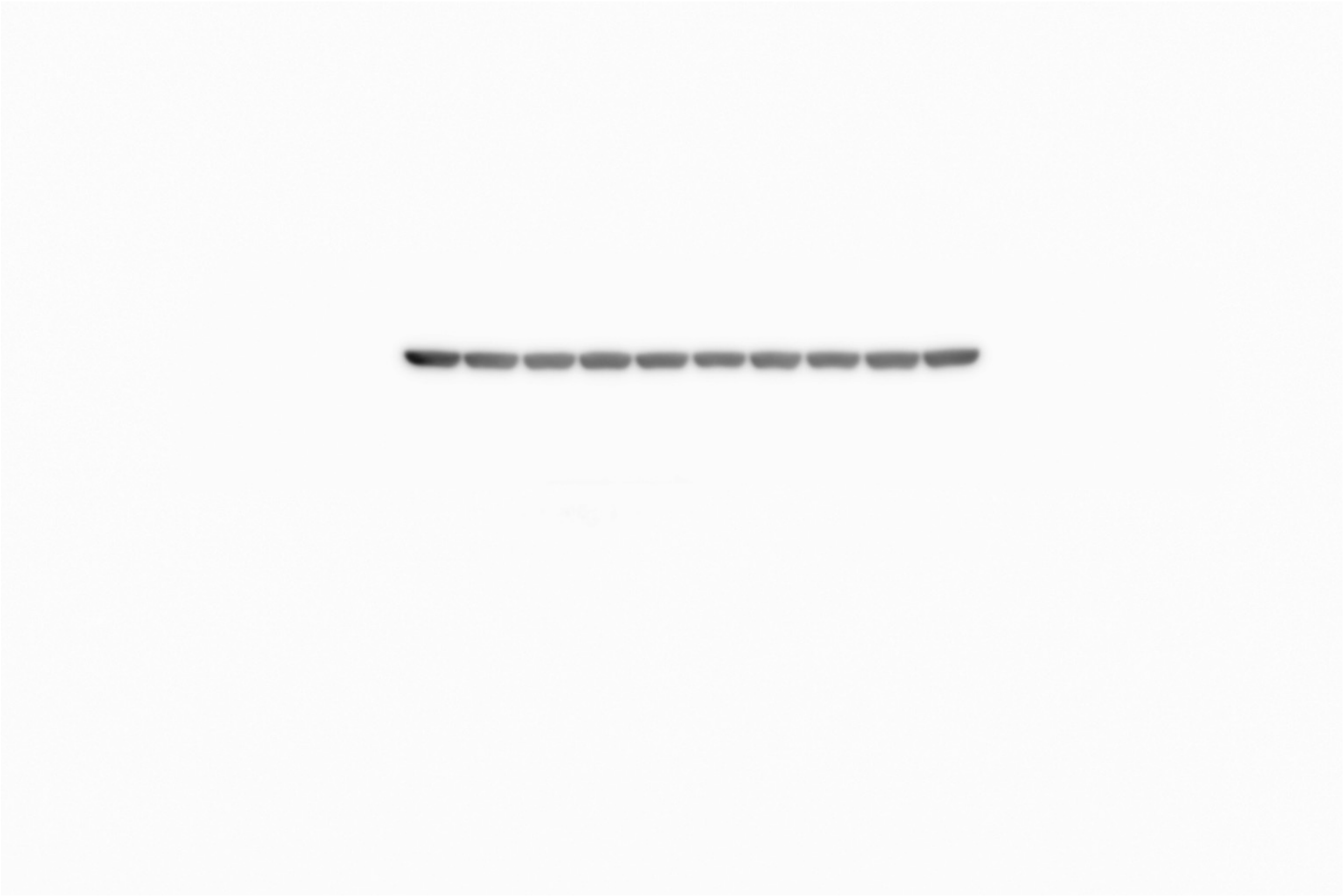

Full and uncropped western blot for Figure 3a
20w HA-LOTUS and LOTUS

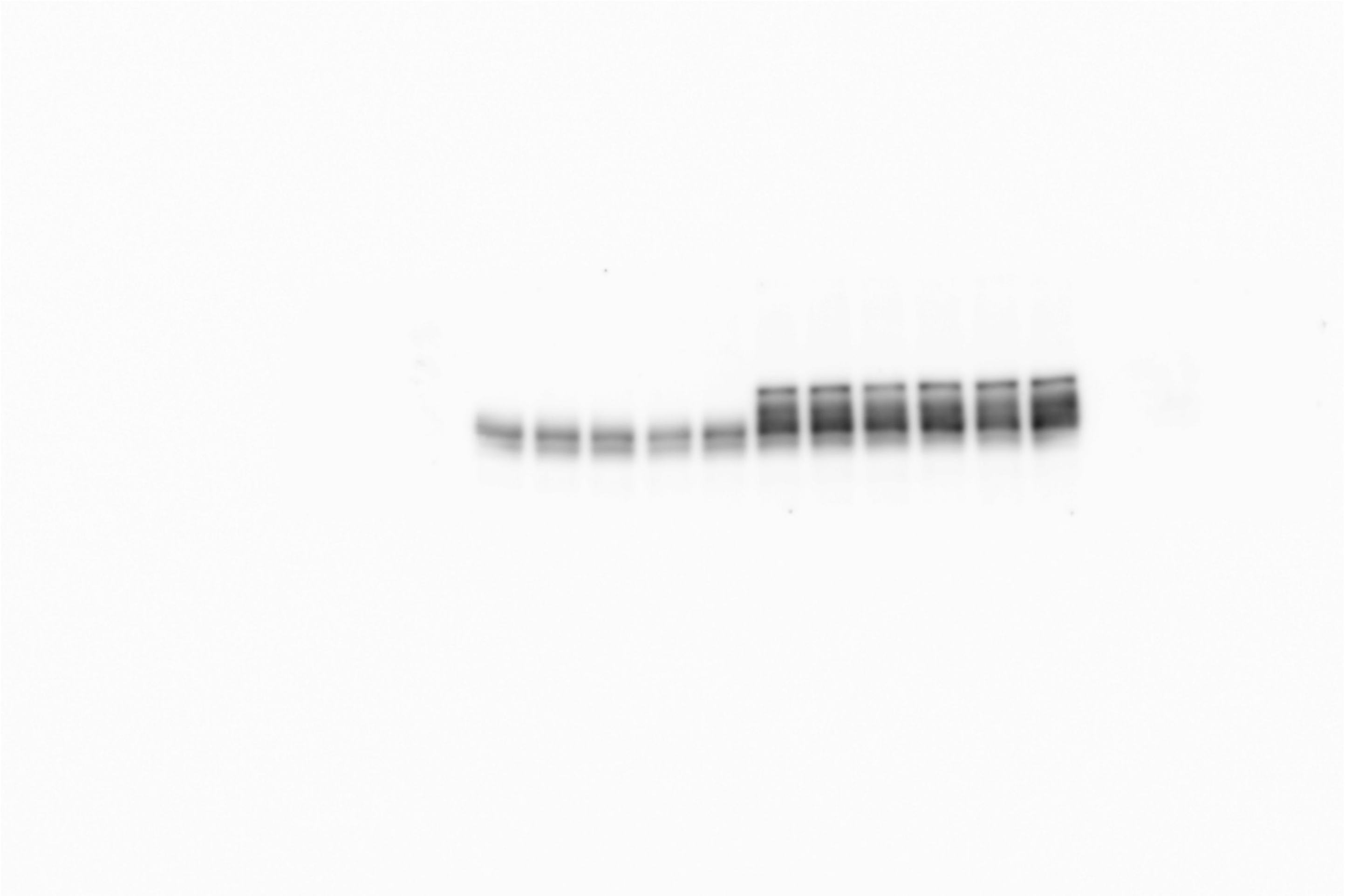

Full and uncropped western blot for Figure 3a
20w β-actin

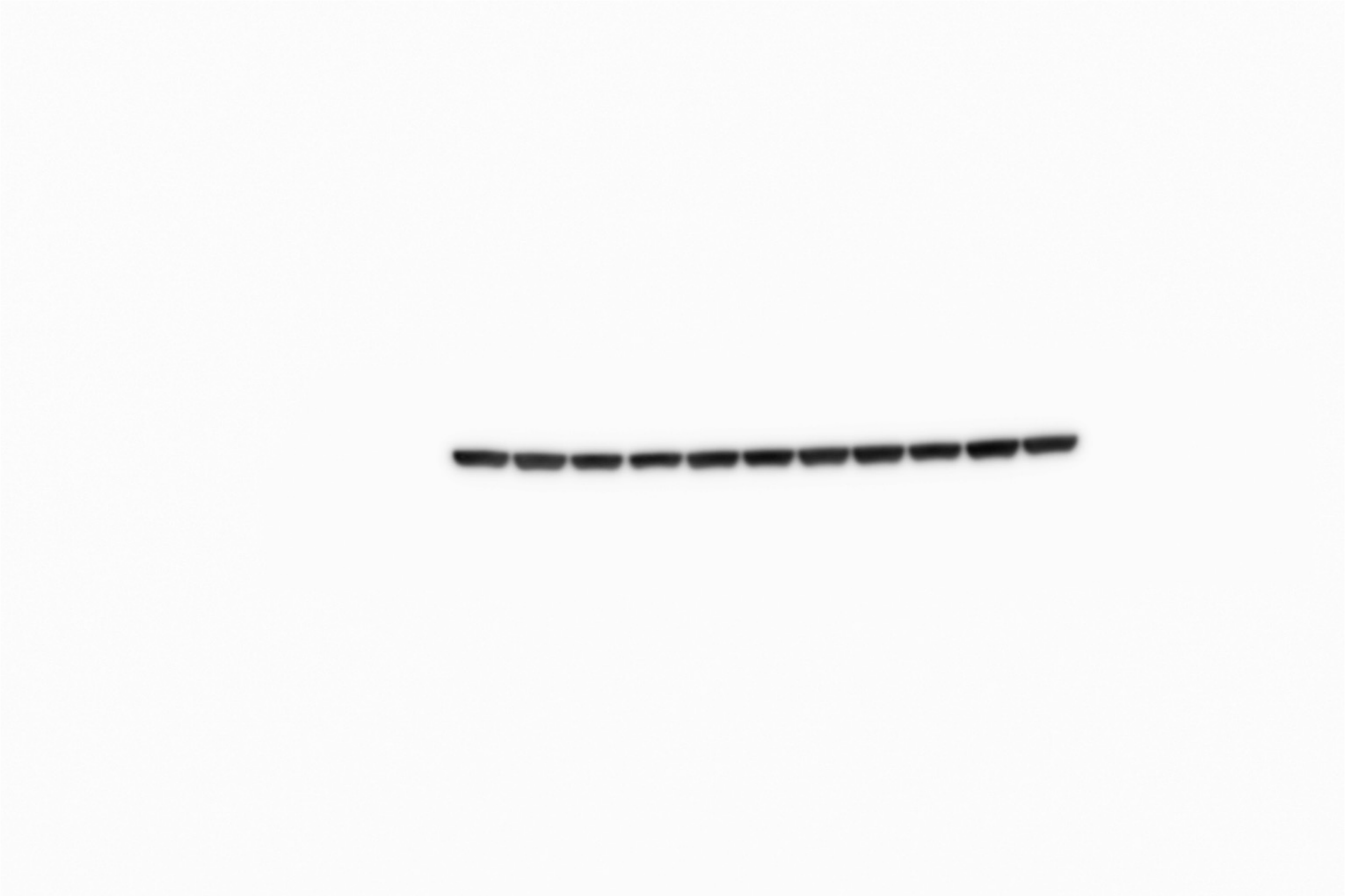

Full and uncropped western blot for Figure 3a
24w HA-LOTUS and LOTUS

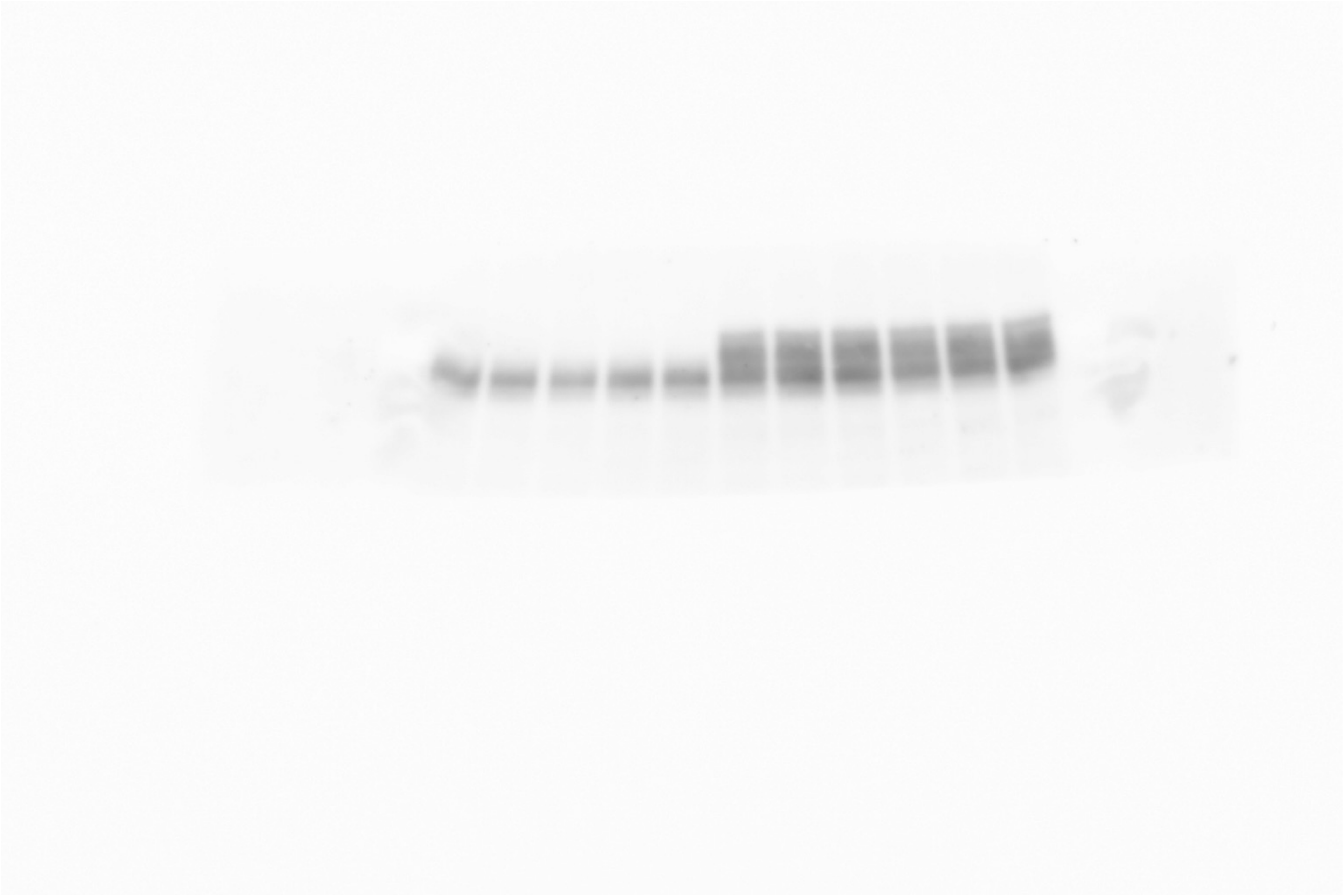

Full and uncropped western blot for Figure 3a
24w β-actin

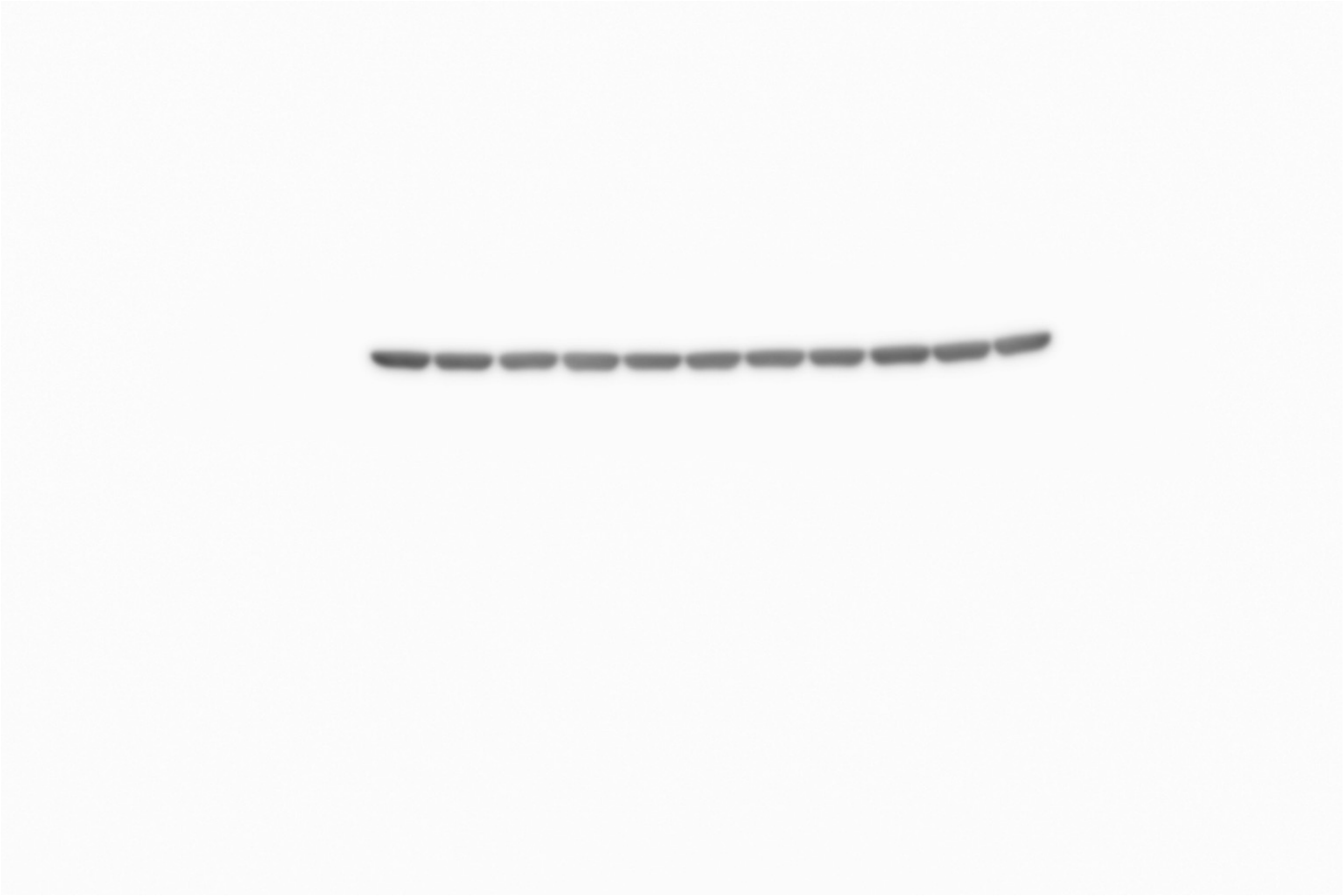

Full and uncropped western blot for Figure 6a
ROCK2

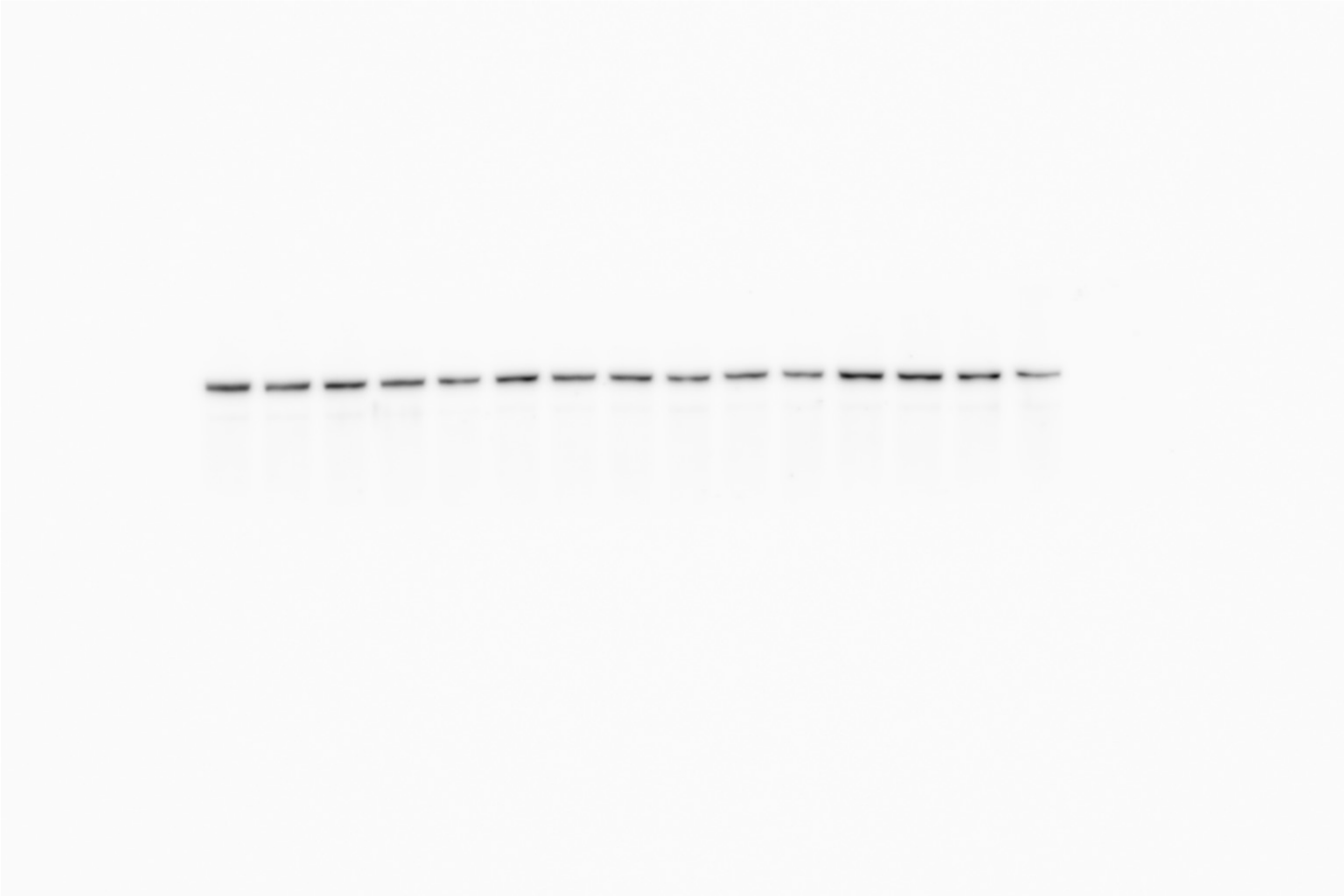

Full and uncropped western blot for Figure 6a
β-actin

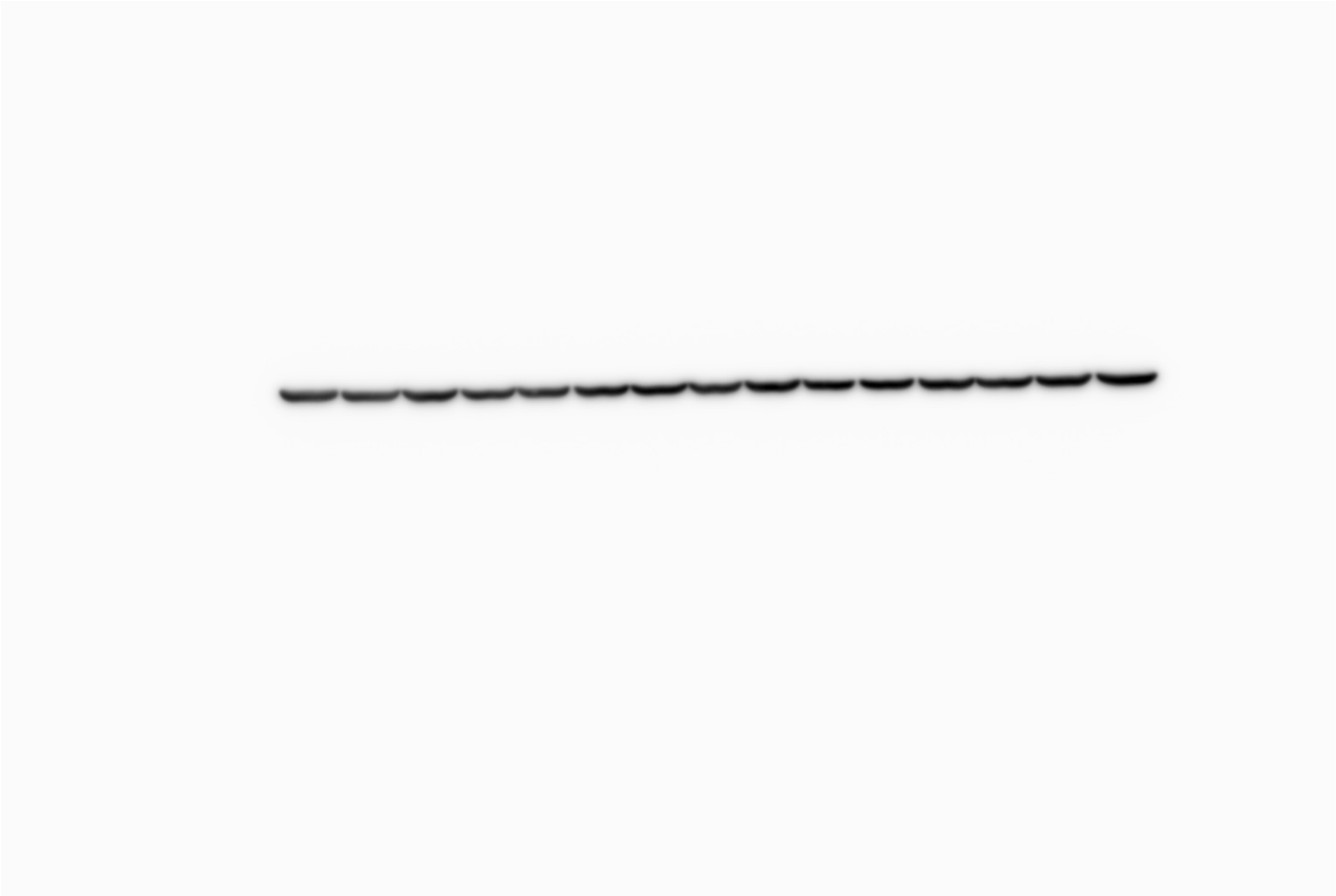

Full and uncropped western blot for Figure 6c
p-LIMK

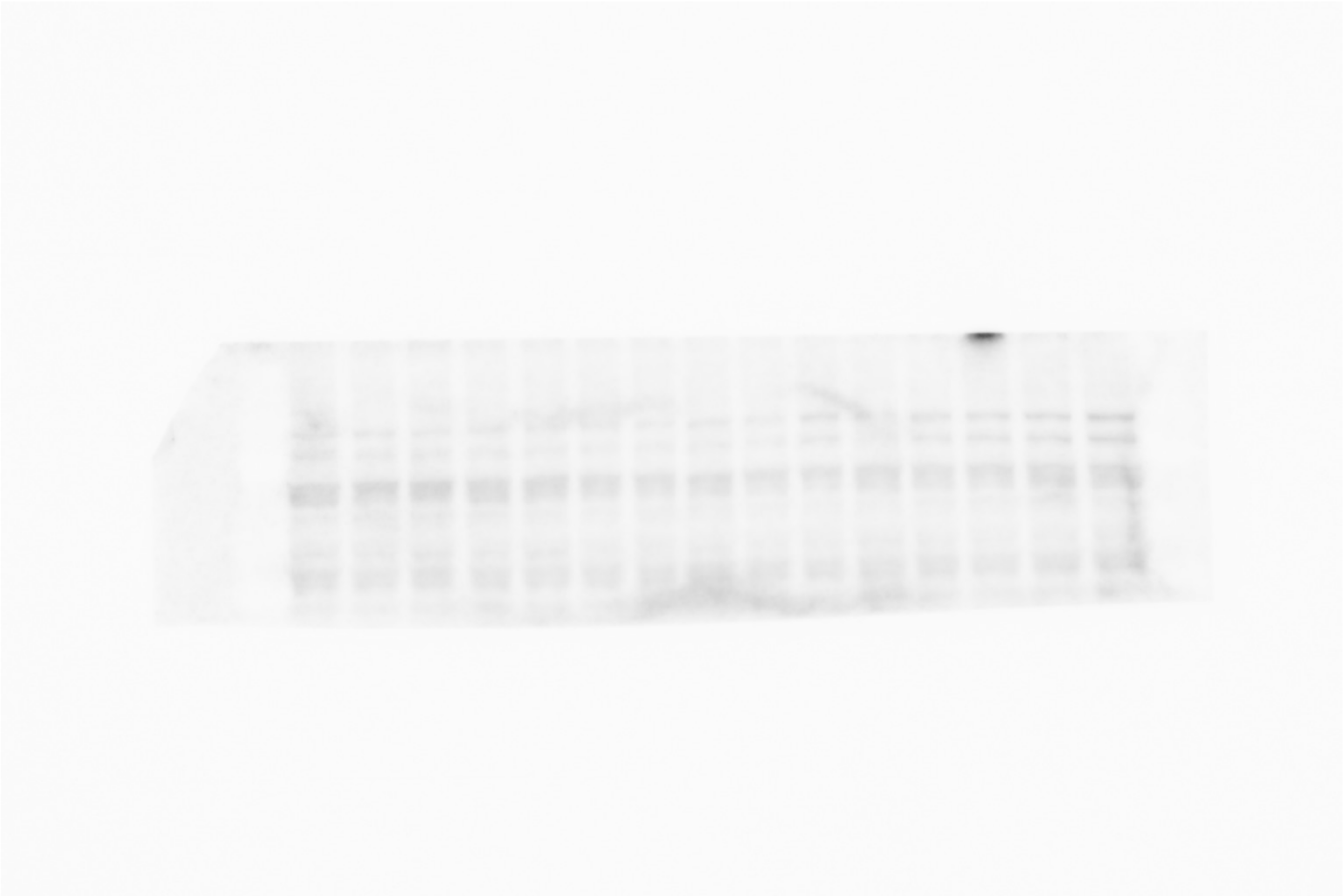

Full and uncropped western blot for Figure 6c
β-actin

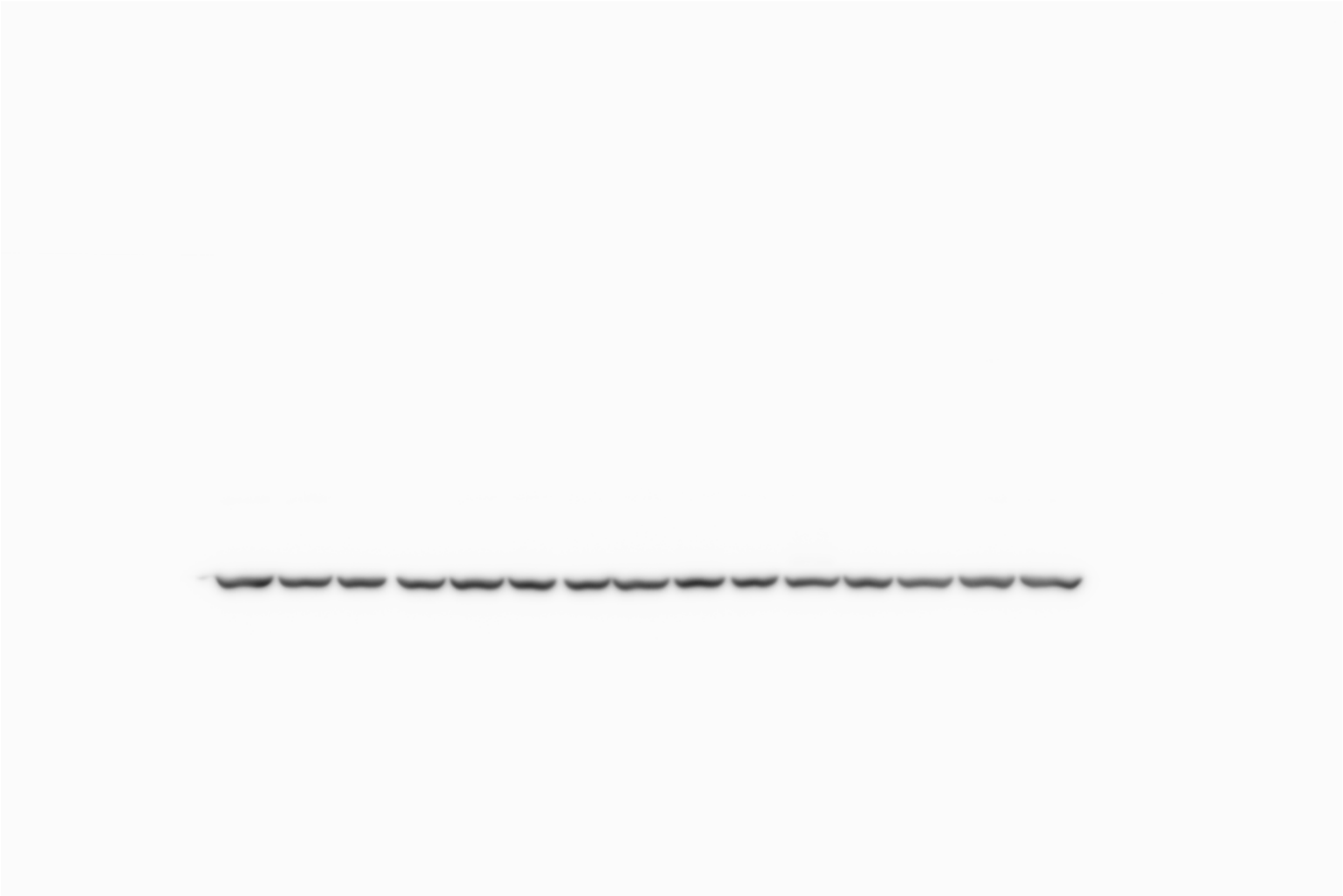

Full and uncropped western blot for Figure 6e
p-cofilin

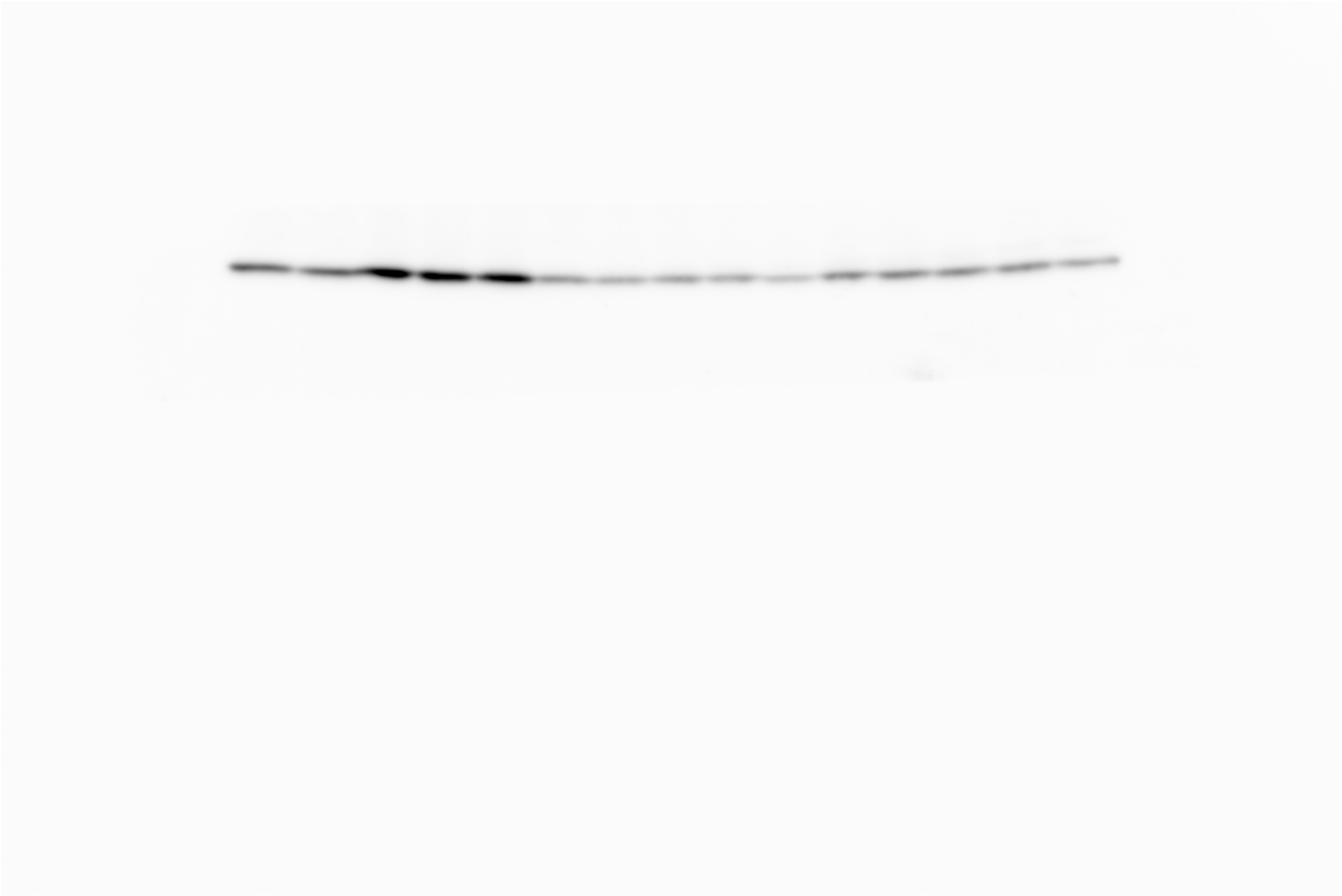

Full and uncropped western blot for Figure 6e
cofilin

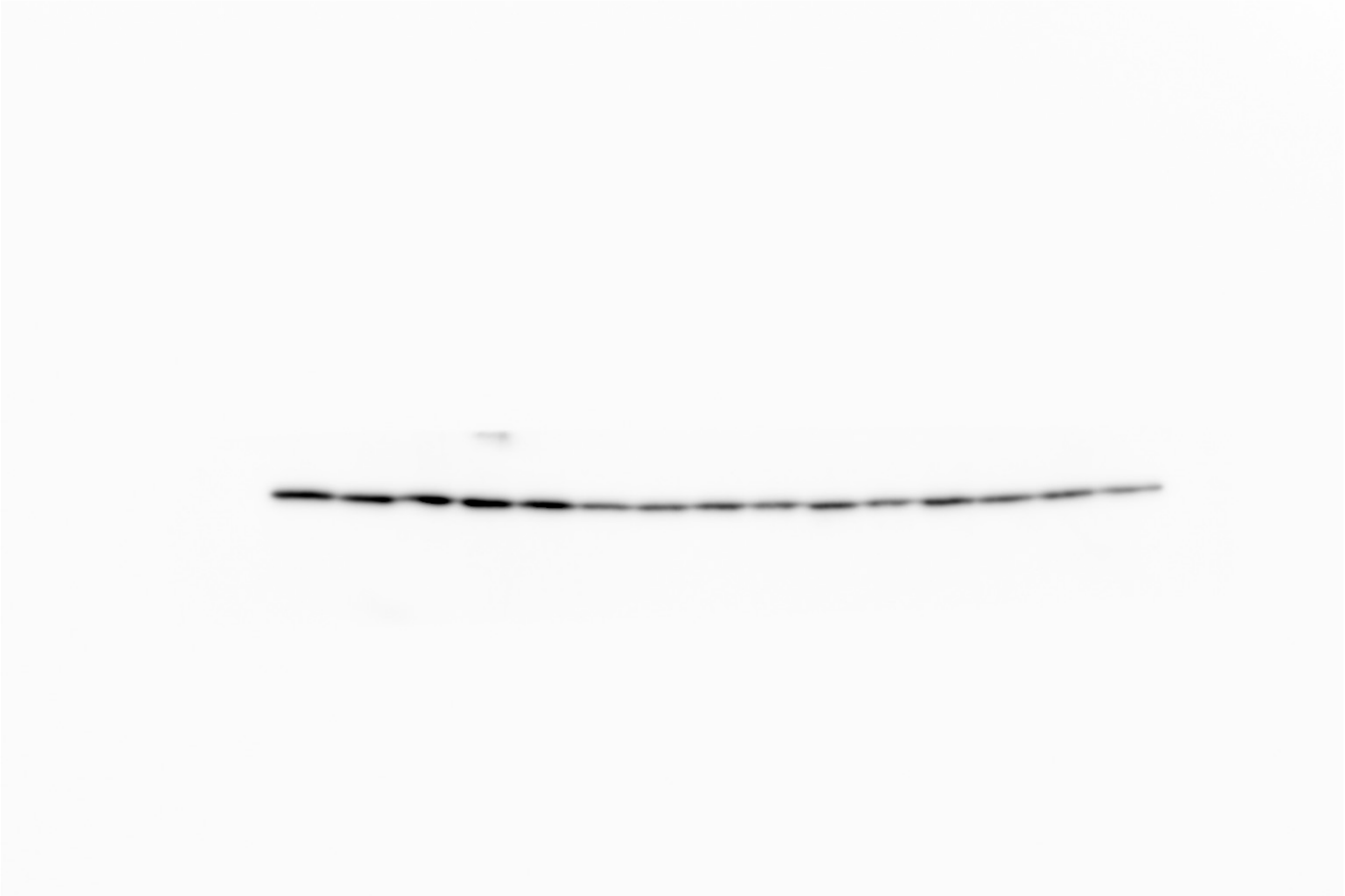

Full and uncropped western blot for Figure 6e
β-actin

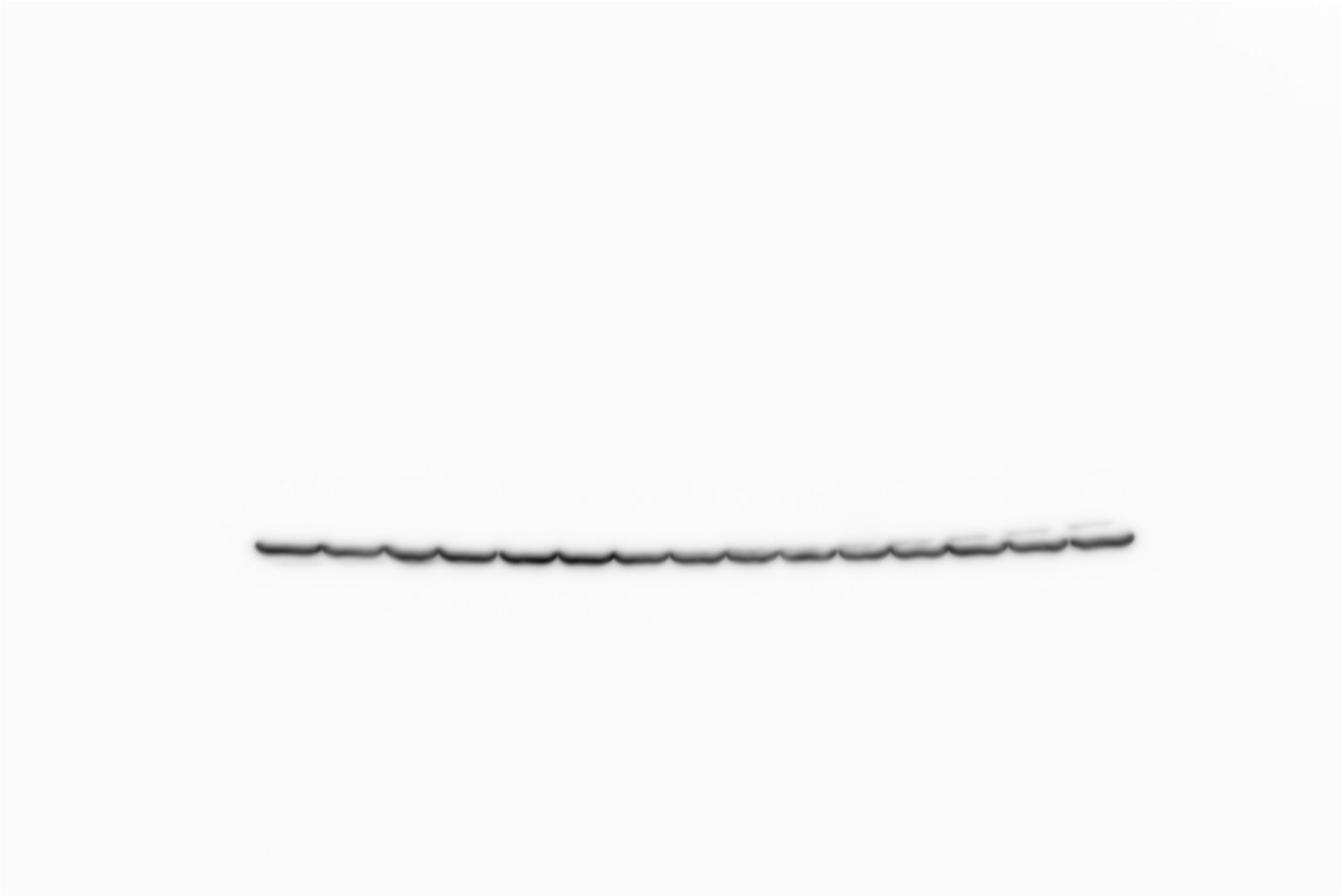

Full and uncropped western blot for Figure 6g
Ataxin-2

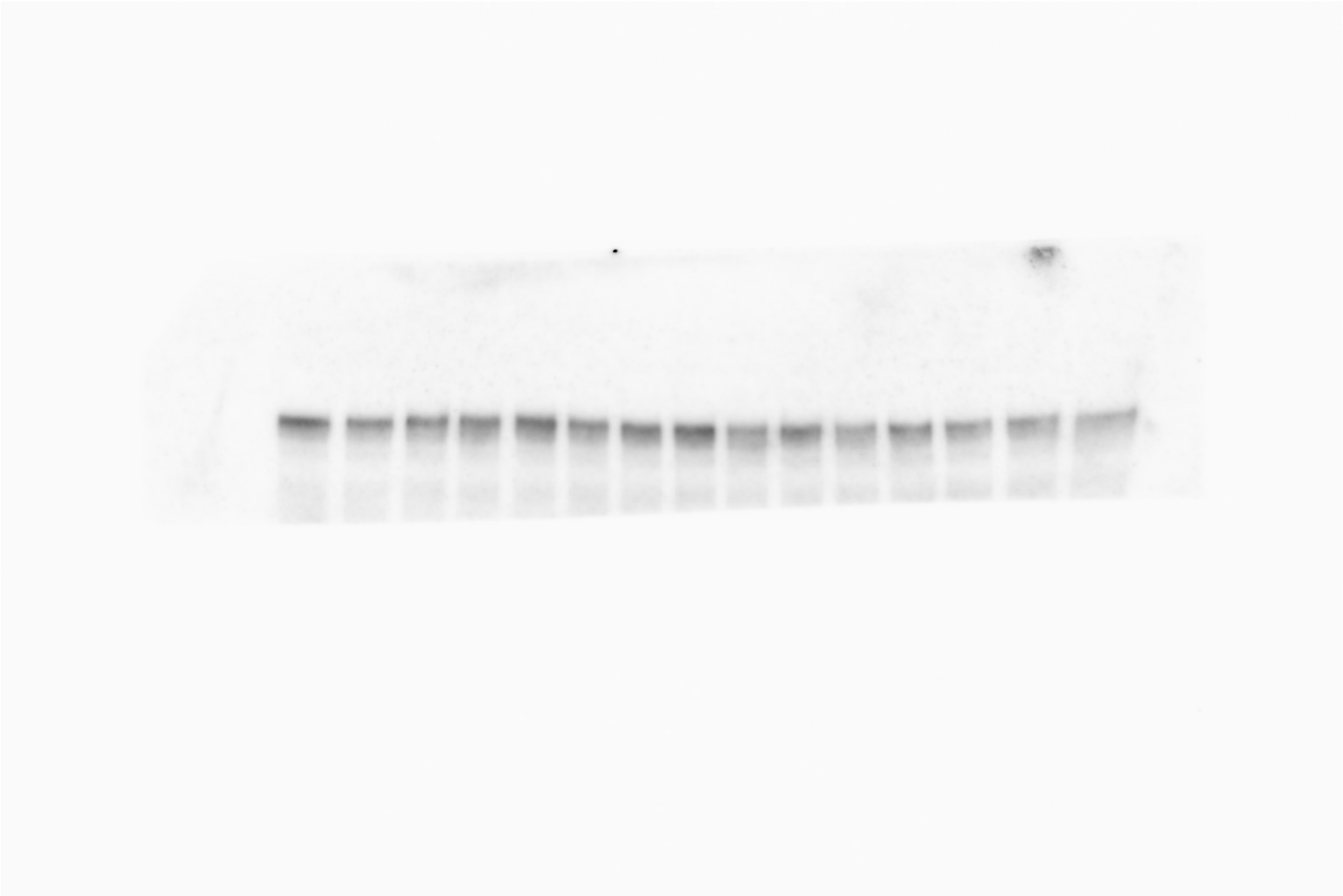

Full and uncropped western blot for Figure 6g
β-actin

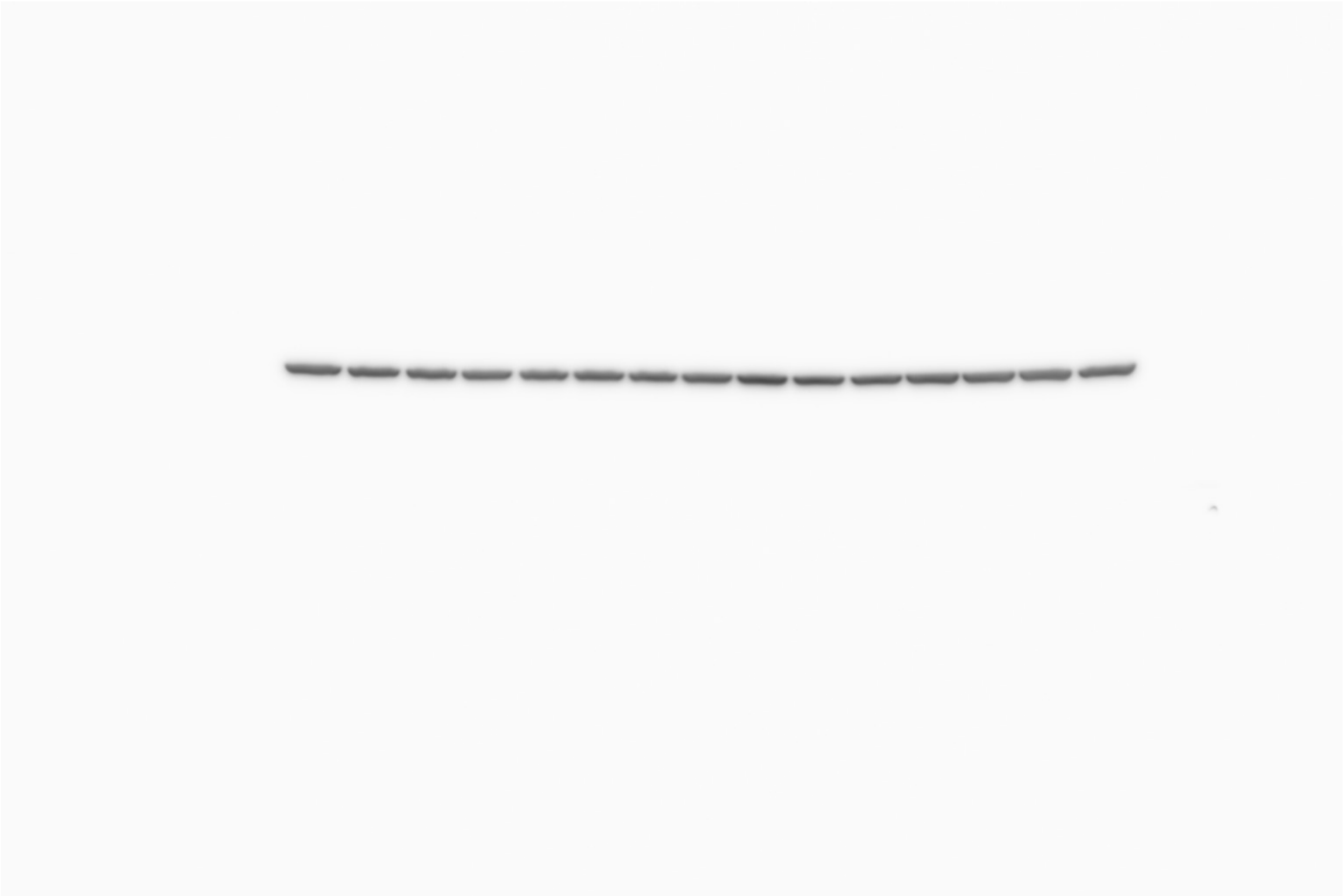

Supplement: Supplementary file 5 — Uncropped WB [file 41420_2023_1758_MOESM5_ESM.pdf]
